# Emerging MXenes for Functional Memories

**DOI:** 10.1002/smsc.202100006

**Published:** 2021-05-05

**Authors:** Yue Gong, Xuechao Xing, Yan Wang, Ziyu Lv, Ye Zhou, Su-Ting Han

**Affiliations:** ^1^ Institute of Microscale Optoelectronics Shenzhen University Shenzhen 518060 P. R. China; ^2^ Institute for Advanced Study Shenzhen University Shenzhen 518060 P. R. China

**Keywords:** functional memories, memristors, MXenes, surface terminations, transistors, work functions

## Abstract

MXenes are a rapidly growing family of 2D materials. The composition, morphology, structure, surface chemistry, and structural configuration of MXenes directly affect their electrochemical performance. For example, when used as the source and drain electrodes in a transistor, MXenes provide increased chemically active interfaces, reduced ion diffusion length, and improved inplane carrier/charge transport kinetics, thus improving the storage performance of the transistor significantly. The work function and bandgap of MXenes can be controlled by tailoring their structure and surface functional groups. This makes MXenes as the first choice for several storage applications. Herein, the latest progress in the synthesis and properties of MXenes is summarized, and it is aimed to boost the motivation of researchers for developing novel MXenes and improving their applications. It emphasizes on the importance of MXenes in high‐density data storage applications, such as flash memories and memristors. The applications of MXenes in artificial synapses, neurons, and logic circuits are also discussed. A future roadmap is outlined for realizing the storage applications of MXenes.

## Graphical abstract

The latest progress in the synthesis and properties of MXenes is summarized. This review emphasizes on the importance of 2D MXenes in energy storage applications, including memristors and field‐effect transistor devices. The applications of MXenes in artificial synapses, neurons, and logic circuits are also discussed, which offers a potential roadmap for realizing the storage applications.

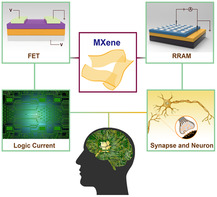

## 1 Introduction

**Figure 1:**
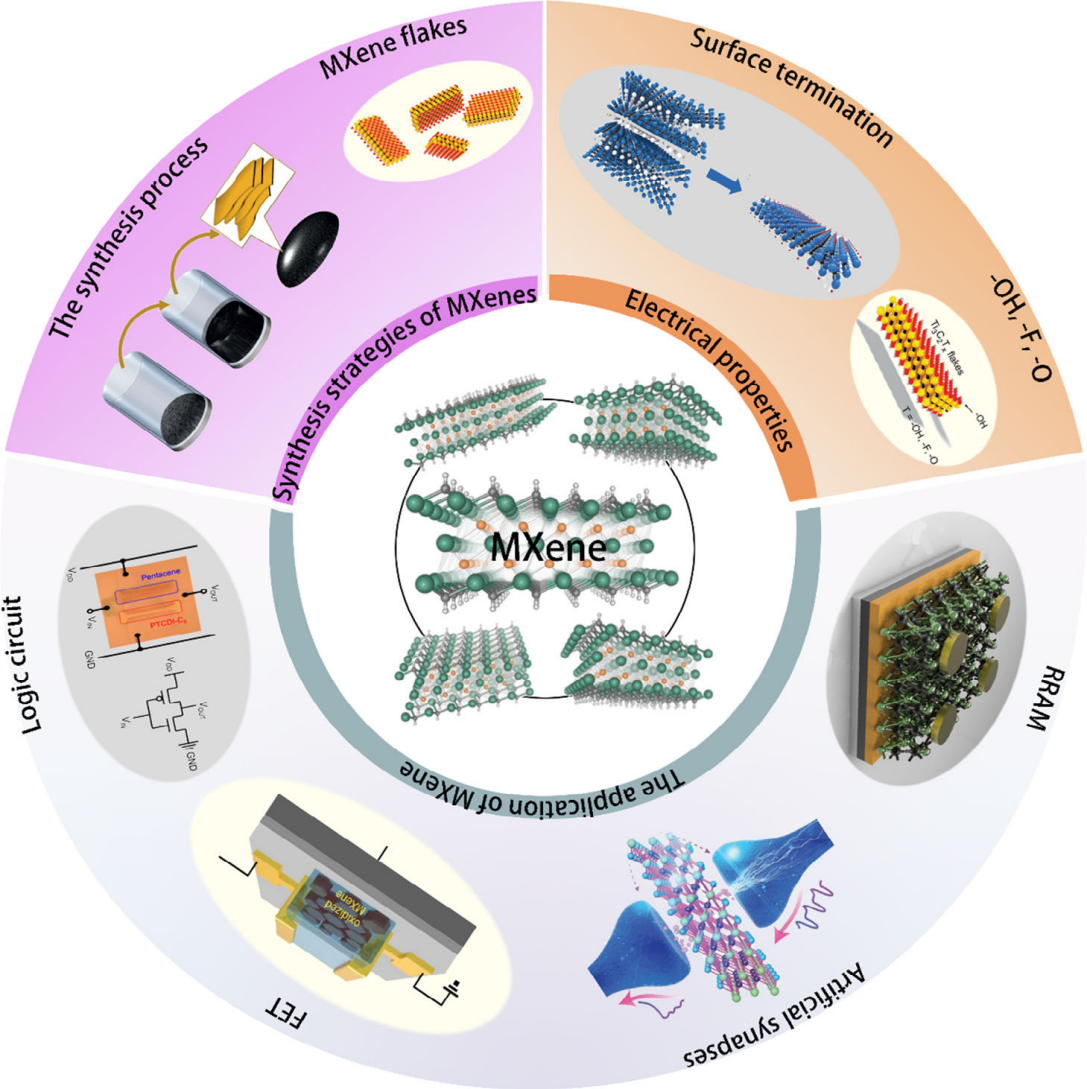
The investigation of MXene synthesis and its electrical properties has inspired MXene‐based RS devices, memory transistors, and optoelectronic applications, such as artificial synapses, neurons, and logic gate applications. Mxene: Reproduced with permission.^[^ ^39^ ^]^ Copyright 2018, Wiley‐VCH. The synthesis process: Reproduced with permission.^[^ ^25^ ^]^ Copyright 2014, Springer Nature. Mxene flakes and —OH, —F, —O: Reproduced with permission.^[^ ^43^ ^]^ Copyright 2016, Wiley‐VCH. Surface termination: Reproduced with permission.^[^ ^40^ ^]^ Copyright 2017, Wiley‐VCH. RRAM: Reproduced with permission.^[^ ^52^ ^]^ Copyright 2019, Elsevier. Artificial synapses: Reproduced with permission.^[^ ^57^ ^]^ Copyright 2021, Wiley‐VCH. FET: Reproduced with permission.^[^ ^21a^ ^]^ Copyright 2020, Wiley‐VCH. Logic circuit: Reproduced with permission.^[^ ^46^ ^]^ Copyright 2019, American Chemical Society.

Since the first demonstration of stable 2D atomic carbon layer graphene, 2D materials, which exhibit significant advantages over bulk materials, have gained immense attention from researchers in various fields.^[^ ^1^ ^]^ However, the gapless band structure, challenging chemical modification, and permanent element and crystal structure of graphene limit its applications to a certain extent.^[^ ^2^ ^]^ In recent years, with the tremendous advancement in synthesis technology, various 2D materials apart from graphene have been developed.^[^ ^3^ ^]^ These materials exhibit high aspect ratio and a thickness of a few atomic layers. One of the newest members of the 2D material family is transition metal carbides and carbonitrides, called MXenes.^[^ ^4^ ^]^ The development and application of MXenes is still a topic of interest for researchers.^[^ ^5^ ^]^ MXenes (MAX phases) have the following chemical composition: M_*n*+1_AX_*n*_, where M is an early transition metal, A is an element of group IIIA or VIIIA, X is C and/or N, and *n* = 1.^[^ ^6^ ^]^ MXenes are formed by selectively etching the A layer from the MAX phase.^[^ ^5^, ^7^ ^]^ The M—X bond primarily exhibits mixed covalent/metal features in the largest stage of the bond.^[^ ^8^ ^]^ As the M—A bond is metallic, it is not easy to mechanically sever the bond between the MAX layers. On the other hand, as the M—A bond is weaker than the M—X bond, it can be severed using aqueous solutions containing fluoride ions, such as aqueous hydrofluoric acid (HF), a mixture of lithium fluoride and hydrochloric acid, or ammonium bifluoride.^[^ ^9^ ^]^ The broken M—A bonds are subject to the spontaneous formation of surface functional groups (denoted as T_*x*_, consisting mainly of =O, —OH, —F groups), hence the general chemical formula M_*n*+1_X_*n*_T_*x*_ is widely used for MXenes. Experimentally, the ratio of different functional groups on the surface of MXene is uncertain and depends on the etching conditions. The chemical properties of MXenes endow them with many interesting mechanical, electronic, magnetic, and electrochemical properties.^[^ ^10^ ^]^ In particular, the strong versatility of MXenes, combined with their 2D morphology and layered structure, makes it simple for MXenes to form composites with other materials.^[^ ^11^ ^]^ These composites exhibit the merits of all the constituents. Therefore, MXenes and composite materials based on MXenes have gained immense attention for various applications.^[^ ^12^ ^]^ MXenes show a peculiar combination of metal conductivity and hydrophilicity of their surface terminations due to the free electrons of the transition metal carbide or nitride backbone. MXenes are considered promising materials for a wide range of applications including energy storage,^[^ ^13^ ^]^ electromagnetic interference shielding,^[^ ^14^ ^]^ transparent conductors,^[^ ^15^ ^]^ gas and pressure sensors,^[^ ^16^ ^]^ water purification,^[^ ^17^ ^]^ photocatalysis,^[^ ^18^ ^]^ thermoelectrics,^[^ ^19^ ^]^ and plasma^[^ ^20^ ^]^ due to their high electronic conductivity and flexible chemical properties. Recently, the storage applications of MXenes have also been reported.^[^ ^21^ ^]^ Specifically, reports of memristors based on layered MXene 2D nanomaterials have attracted significant attention. Nanofloating gate transistor memory (NFGTM) shows great application prospects in nonvolatile memory due to its advantages of high stability and low operating voltage.^[^ ^22^ ^]^ However, capacitive coupling induced by the parasitic capacitance following charge trapping and low gate coupling ratio are experienced during the miniaturization phase necessary to increase the storage ability of these transistors. Due to their intrinsic 2D structure, MXenes can overcome these shortcomings. MXenes offer a wide range of Ohmic contacts due to their work feature tunability, which can improve their carrier injection performance.^[^ ^23^ ^]^ These characteristics make MXenes promising electrode materials for field‐effect transistors (FETs). Pulse voltage is used to simulate the action of human synapses, which can realize the change in resistance to study the characteristics of memristors. Interestingly, MXenes are also used to fabricate high‐performance logic circuits and artificial synapses as active media. The low power consumption and fast switching of MXene memory devices render them suitable for energy‐saving neuromorphic computing, as shown in **Figure** **1**. This Review provides a brief overview of the latest advances in MXene memory and its practical applications. The Review also discusses the synthesis methods used for preparing MXenes and the effect of the synthesis method on the performance of MXenes. Furthermore, the electronic properties, topological properties, and work function (WF) of MXenes, as well as the application potential of devices are discussed. In addition, this Review discusses the possibilities of utilizing MXenes for specific device applications, such as organic field‐effect transistors (OFETs), nonvolatile memory, artificial synapses and neurons, and logic gate circuits. Finally, the problems faced by memory devices based on MXenes and their practical solutions are outlined to encourage further research on MXenes. This Review can be used as an important benchmark to promote research on novel 2D materials to realize their storage applications.

## 2 Synthesis Strategies for MXenes

It can be said that the most studied independent 2D material is graphene, which is a single‐layer material that was produced by mechanical peeling in 2004. The applications of graphene range from supercapacitor electrodes to reinforced composite materials. While graphene has gained more attention than all other 2D materials, its simple chemical properties and weak interlayer van der Waals bonds restrict its application in multilayer structures. New characteristics can be generated by developing complex layered structures comprising more than one element because they provide more compositional variables that can be tuned to achieve the desired characteristics. To date, various 2D materials including graphene, transition metal disulfides (TMDs), layered double hydroxides, silylene, and black phosphorene have been discovered and extensively studied. Transition metal carbides, nitrides, and carbonitrides (MXenes) were first reported in 2011 by the Gogotsi team as a new member of the 2D materials family.^[^ ^24^ ^]^ The preparation of high‐quality MXenes is difficult because of the agglomeration tendency of its 2D nanosheets. Briefly, the preparation strategies of MXenes can be classified as top‐down and bottom‐up synthesis strategies. The former approach is based on the exfoliation of bulk crystals into few layer or monolayer sheets, whereas the latter is the reverse process, in which the desired material is developed from atoms or molecules. Finally, to obtain single‐layer MXene, the delamination of multilayer MXenes is the most crucial step after the synthesis. In this Review, we concentrate primarily on the top‐down wet etching method as it is the most commonly used technique for processing MXenes.

### 2.1 Top‐Down Synthesis

**Figure 2:**
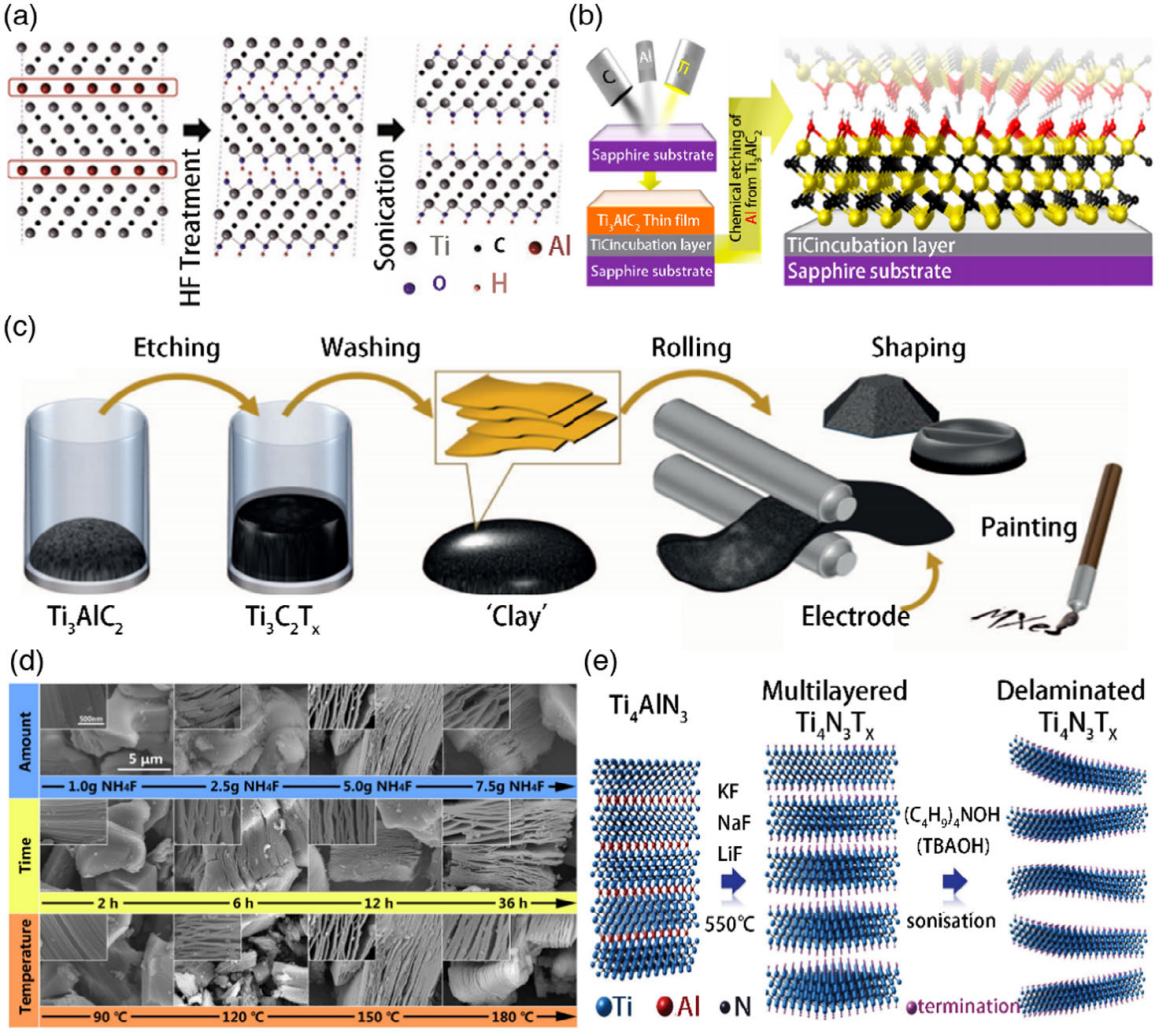
a) Schematic of exfoliation process for Ti_3_AlC_2_ peeling process. The key processes include the replacement of aluminum atoms with hydroxyl groups after a hydrogen fluoride reaction, the breakdown of hydrogen bonds, and the separation of nanosheets after ultrasonic methanol treatment. b) The image on the left shows the magnetron sputtering of Ti, Al, and C on a sapphire substrate to form a few nanometers of TiC incubation layer and then deposit Ti_3_AlC_2_. A schematic diagram of hydroxyl‐terminated Ti_3_AlC_2_ after selective etching of Al from Ti_3_AlC_2_ is shown in the image on the right. c) Schematic diagram of MXene clay synthesis and electrode preparation. The largest phase is etched in a solution of acid and fluoride salt, then washed with water to remove the reaction products. d) Field emission scanning electron microscopy image of Ti_3_AlC_2_ under various conditions before and after NH_4_F therapy. It can be observed from the figure that, with a lower amount of NH_4_F and a shorter time, the response between Ti_3_AlC_2_ and NH_4_F is pretty weak. e) Schematic of the Ti_4_N_3_T_*x*_ synthesis. At 550 °C under argon, the Ti_4_AlN_3_ is treated with molten salt, and then the multilayer MXene is layered with TBAOH. a) Reproduced with permission.^[^ ^24^ ^]^ Copyright 2011, Wiley‐VCH. b) Reproduced with permission.^[^ ^26^ ^]^ Copyright 2014, American Chemical Society. c) Reproduced with permission.^[^ ^25^ ^]^ Copyright 2014, Springer Nature. d) Reproduced with permission.^[^ ^27^ ^]^ Copyright 2016, Springer Nature. e) Reproduced with permission.^[^ ^28^ ^]^ Copyright 2016, Royal Society of Chemistry.

As shown in **Table** **1** and **2**, more than 30 different MXenes have been synthesized by chemically etching specific atomic layers from layered carbide, nitriding, or carbonitriding the precursors. The etchants used for this purpose can mainly be divided into two categories: HF and fluoride salts. HF etching is the most widely used method to manufacture Ti_3_C_2_T_*x*_. As shown in **Figure** **2**a, Naguib et al. first reported a few Ti_3_C_2_ layers synthesized by selectively etching Al from Ti_3_AlC_2_ in HF acid at room temperature.^[^ ^24^ ^]^ By varying the reaction temperature from room temperature to 55 °C and by controlling the reaction time and HF concentration, HF etching can produce various MXenes. HF is a highly selective standardized etchant, and the etching reaction occurs as follows

(1) $$M_{n+1}{\text{AX}}_{n}+3\text{HF}(\text{or }4\text{HF})\to M_{n+1}X_{n}+{\text{AF}}_{3}({\text{or AF}}_{4})+3/2H_{2}(\text{or }2H_{2})$$

(2) $$M_{n+1}X_{n}+2H_{2}O\to M_{n+1}X_{n}{\left(\text{OH}\right)}_{2}+H_{2}$$

(3) $$M_{n+1}X_{n}+2\text{HF}\to M_{n+1}X_{n}F_{2}+H_{2}$$

**Table 1:** Etching with HF

MXene	Precursor	Etchant	Time [h]	*T* [°C]	Ref.
Ti_2_CT_*x*_	Ti_2_AlC	10%HF	10	RT	[63]
V_2_CT_*x*_	V_2_AlC	50%HF	90	RT	[64]
Nb_2_CT_*x*_	Nb_2_AlC	50%HF	90	RT	[65]
Mo_2_CT_*x*_	Mo_2_Ga_2_C	25%HF	160	55	[64]
(Ti,V)_2_CT_*x*_	(Ti,V)_2_AlC	50%HF	19	RT	[66]
(Ti,Nb)_2_CT_*x*_	(Ti,Nb)_2_AlC	50%HF	28	RT	[67]
Ti_3_C_2_T_*x*_	Ti_3_AlC_2_	50%HF	2	RT	[24]
Ti_3_C_2_T_*x*_	Ti_3_AlC_2_	10%HF	24	RT	[24]
(Ti,V)_3_C_2_T_*x*_	(Ti,V)_3_AlC_2_	50%HF	18	RT	[66]
Ti_3_CNT_*x*_	Ti_3_Al(C,N)_2_	30%HF	18	RT	[67]
Zr_3_C_2_T_*x*_	Zr_3_Al_3_C_2_	50%HF	72	RT	[24]
(Cr,V)_3_C_2_T_*x*_	(Cr,V)_3_AlC_2_	50%HF	69	RT	[68]
Mo_2_TiC_2_T_*x*_	Mo_2_TiAlC_2_	50%HF	48	55	[67]
Nb_4_C_3_T_*x*_	Nb_4_AlC_3_	50%HF	90	RT	[69]
(Nb,Ti)_4_C_3_T_*x*_	(Nb,Ti)_4_AlC_3_	50%HF	90	50	[70]
Ta_4_C_3_T_*x*_	Ta_4_AlC_3_	50%HF	72	RT	[67]
Mo_2_Ti_2_C_3_T_*x*_	Mo_2_Ti_2_AlC_3_	50%HF	96	55	[67]
Ti_2_NT_*x*_	Ti_2_AlN	5%HF	24	RT	[64]
V_4_C_3_T_*x*_	V_4_AlC_3_	40%HF	165	RT	[71]
Mo_4/3_CT_*x*_	(Mo_2/3_Y_1/3_)_2_AlC	48%HF	60	RT	[72]
W_4/3_CT_*x*_	(W_2/3_Sc_1/3_)_2_AlC	48%HF	30	RT	[73]
Hf_3_C_2_T_*x*_	Hf_3_[Al (Si)]_4_C_6_	35%HF	60	RT	[74]

**Table 2:** Fluoride‐based etching

MXene	Precursor	Etchant	Time [h]	*T* [°C]	Ref.
Ti_3_C_2_T_*x*_	Ti_3_AlC_2_	3 m LiF + 6 m HCl	45	40	[25]
Ti_2_CT_*x*_	Ti_2_AlC	0.9 m LiF + 6 m HCl	15	40	[75]
Mo_2_CT_*x*_	Mo_2_Ga_2_C	3 m LiF + 12 m HCl	384	35	[76]
V_2_CT_*x*_	V_2_AlC	2 g LiF + 40 m HCl	48	90	[77]
Ti_3_CNT_*x*_	Ti_3_AlCN	0.66 g LiF + 6 m HCl	12	35	[78]
Cr_2_TiC_2_T_*x*_	Cr_2_TiAlC_2_	5 m LiF + 6 m HCl	42	55	[69]
(Nb_0.8_Zr_0.2_)_4_C_3_T_*x*_	(Nb_0.8_Zr_0.2_)_4_AlC_3_	LiF + 12 m HCl	168	50	[79]
W_4/3_CT_*x*_	(W_2/3_Sc_1/3_)_2_AlC	4 g LiF + 12 m HCl	48	35	[73]
Mo_2_Ga_2_C	Mo_2_CT_*x*_	15 m LiF + 9 m HCl	75	32	[79]
Ti_3_C_2_T_*x*_	Ti_3_AlC_2_	1 m NH_4_HF_2_	12	80	[26]
Ti_3_C_2_T_*x*_	Ti_3_AlC_2_	1 m NH_4_HF_2_	120	RT	[80]
Ti_3_C_2_T_*x*_	Ti_3_AlC_2_	5 g NH_4_F	24	150	[27]
Ti_3_C_2_T_*x*_	Ti_3_AlC_2_	30% NH_4_OH	18	RT	[26]
Ti_3_C_2_T_*x*_	Ti_3_AlC_2_	NH_3_F	20	150	[27]
Ti_4_N_3_T_*x*_	Ti_4_AlN_3_	59%KF + 29%LiF + 12%NaF	0.5	550	[28]

The etching conditions for the largest phase containing Al differ depending on the transition metal, material composition, atomic bonds, and particle size. Compared with the covalent/metal/ion mixed M—X bond, the weaker M—A metal bond is the key to the feasibility of selectively extracting the “A” atomic layer from the ternary maximum phase precursor. As shown in Table 1, HF has been used for preparing more than 30 MXenes. During the peeling process, the element reacts with high frequency due to its lower bond energy to form fluorides (e.g., AlF_3_, SiF_4_) and gaseous hydrogen (H_2_), resulting in a fold‐like structure of M_*n*+1_X_*n*_. Experimental results have shown that an increase in the number of M atoms requires a longer etching time and stronger etching strength. This may be related to the M—Al bond. As the M—Al bond is metallic, we speculate that more the number of M valence electrons, stronger is the etching required. In this process, the yield, defects, particle size, and ratio of surface termination groups of MXenes may strongly depend on the etching conditions (HF concentration, reaction temperature, and etching time). It is worth noting that the synthesis conditions of one MXene should not be extended to other MXenes, because each MAX has its own stability and reactivity to a different etchant. Generally speaking, M_4_AX_3_ (413 phase) requires stronger etching conditions compared with M_3_AX_2_ (312 phase) and M_2_AX (211 phase). The aggressive etching conditions (high HF concentration, high temperature, long etching time) may induce overetching which leads to formation of defects in the M position or complete removal of both A and M elements. Therefore, the compromise should be taken between etching and maximum stability to obtain 2D MXenes with the optimum performance. Recently, a review has been carried out on the wet acid etching of MXenes. It should be noted that the metal bonds hanging on the surface after selective etching are unstable and can react spontaneously with the aqueous solution and change the surface ends such as O, —OH, and —F (Equation (2) and (3)). In the wet acid etching process, the alteration of the surface ends is inevitable. This alteration renders the surface hydrophilic, and hence significantly affects the electrical, optical, magnetic, and mechanical properties of the resulting MXenes. MXenes obtained by HF etching have been applied to other MXene phases, such as the Ti_2_CT_*x*_, V_2_CT_*x*_, Nb_2_CT_*x*_, Mo_2_CT_*x*_, (Ti,V)_2_CT_*x*_, (Ti,Nb)_2_CT_*x*_, Ti_3_C_2_T_*x*_, (Ti,V)_3_C_2_T_*x*_, Ti_3_CNT_*x*_, Zr_3_C_2_T_*x*_, (Cr,V)_3_C_2_T_*x*_, Mo_2_TiC_2_T_*x*_, Ti_2_NT_*x*_, V_4_C_3_T_*x*_, Mo_4/3_CT_*x*_, W_4/3_CT_*x*_, and Hf_3_C_2_T_*x*_ phases.

HF acid is a dangerous substance that can cause permanent damage to eyes, skin, and bones. Therefore, considering the hazards of HF acid to the human body and environment, it is imperative to develop other harmless corrosives. Hydrochloric acid (HCl) and LiF react in situ to form HF, which selectively etches A atoms. Recently, similar etching results have been obtained using a combination of HCl and LiF, suggesting that the presence of protons and fluoride ions is a sufficient condition for the etching and formation of MXene “clay” (Table 2). To dissolve aluminum and remove the 2D carbide sheet, Ghidiu et al. developed a relatively safe process involving the reaction between HCl, which is very cheap and easily available, and a fluoride salt, as shown in Figure 2c.^[^ ^25^ ^]^ They used 6 m HCl to dissolve 3 m LiF. Then, Ti_3_AlC_2_ powder was added and the resulting mixture was heated to 40 °C for 45 h to obtain clay‐like Ti_3_C_2_T_*x*_, which could be rolled into thin sheets. The resulting deposits were washed by corrosion to remove the reaction products and increase their pH (several cycles of water, centrifugation, and unloading). Although the HF etching technology has been widely used in the manufacture of Ti_3_C_2_T_*x*_, it requires an additional HF treatment step. This hinders the practical application of this technology. Interestingly, Halim et al. found that ammonium bifluoride (NH_4_HF_2_) containing weakly acidic environment‐friendly fluorine can be used instead of HF to synthesize MXenes.^[^ ^26^ ^]^ This approach prevents the generation of harmful gases. As shown in Figure 2b, this process starts with the sputter deposition of Ti_3_AlC_2_ (the initial formation of the TiC incubation layer). The next step is to etch the Al layer to form a 2D Ti_3_C_2_T_*x*_ layer, where T_*x*_ represents the —O, —OH, or —F end face produced by the HF water etchant. Here, the Ti_3_C_2_ surface is assumed to be OH‐terminated. During the etching of Ti_3_AlC_2_ by NH_4_HF_2_, the etching of aluminum and the insertion of the ammonium layer occur simultaneously. Therefore, it is reasonable to conclude the occurrence of the following reaction in this case

(4) $${\text{Ti}}_{3}{\text{AlC}}_{2}+3{\text{NH}}_{4}{\text{HF}}_{2}={\left({\text{NH}}_{4}\right)}_{3}{\text{AlF}}_{6}+{\text{Ti}}_{3}C_{2}+3/2H_{2}$$

At the same time, it can be observed that the lattice constant *c* of Ti_3_C_2_T_*x*_ corroded by NH_4_HF_2_ is 25% larger than that of Ti_3_C_2_T_*x*_ corroded by HF. Wang et al. reported a simple hydrothermal method to control the morphology of Ti_3_C_2_T_*x*_ by varying the amount of NH_4_, temperature, and hydrothermal reaction time. Figure 2d shows the field‐effect scanning electron microscopy image of Ti_3_AlC_2_ before and after the NH_4_ treatment under different conditions.^[^ ^27^ ^]^ It can be seen from the figure that the reaction between Ti_3_AlC_2_ and NH_4_F was very weak when the amount of NH_4_F was low and the reaction time was short. Therefore, unlike the untreated Ti_3_AlC_2_, the NH_4_F‐treated Ti_3_AlC_2_ showed slight change in its surface morphology. With an increase in the NH_4_F dosage and reaction time, the amount of HF produced by NH_4_F hydrolysis increased, which promoted the etching process of Ti_3_AlC_2_. The Ti_3_C_2_T_*x*_ prepared using this method showed high specific capacitance and low resistance. After 1000 cycles, the capacitance of Ti_3_C_2_T_*x*_ is reduced by only 6%, indicating its good long‐term cycle stability. Therefore, NH_4_F hydrothermal synthesis is a safe and simple method to prepare high‐performance Ti_3_C_2_T_*x*_. Since the development of this method, various similar methods using a combination of fluoride salts (such as NaF, KF, and LiF) and acids as corrosive agents have been successfully developed. In these deuterium generation methods, fluoride salts and strong acids react in situ to generate HF. Interestingly, the reaction between a large number of metal cations (e.g., Li^+^, Na^+^, K^+^, and Ca^2+^) and water can spontaneously generate an aqueous solution of the fluoride salt in the interlayer between the Ti_3_C_2_ layers, leading to the formation of multifunctional products with functionalities such as expanded and modified spacing between the surface adsorption layers. Gogotsi and coworkers successfully carried out the heating of Ti_4_AlN_3_ under an argon (Ar) atmosphere in a molten salt to synthesize Ti_4_N_3_T_*x*_.^[^ ^28^ ^]^ The fluoride salt mixture comprised 59 wt% of KF, 29 wt% of LiF, and 12 wt% of NaF, which corresponds to the ternary eutectic composition. Subsequently, the mixture of Ti_4_AlN_3_ and fluoride salt was heated at 550 °C for 30 min in an alumina crucible under the flow of Ar, and the heating rate was 10 °C min^−1^. The synthesis procedure for Ti_4_N_3_T_*x*_ can be extended to prepare other 2D transition metal nitrides (Figure 2e).

In addition to the MAX phase, MXenes can also be generated from non‐MAX precursors by the selective acid etching of relatively weakly bonded element units instead of a single element. Meshkian et al. prepared an exfoliated 2D Mo_2_C layer via HF etching. They reported for the first time about the etching of MXenes from MAX phase‐related materials without Al and the preparation of Mo‐based MXenes. These findings have opened up the possibility of realizing the applications of Mo‐containing MXene materials and investigating 2D Mo_2_C layers as high‐performance thermoelectric materials. Furthermore, these findings may lead to the development of novel 2D materials based on gallium and related materials as the largest phase. Another non‐MAX phase, Mo_2_Ga_2_C, has been used as the precursor for the synthesis of Mo_2_CT_*x*_. The structure of Mo_2_Ga_2_C is composed of a stack of Mo_2_C layers and a double‐layer Ga structure. Currently, Mo_2_Ga_2_C is used to etch Ga double‐layer films, and both HF and LiF + HCL can be used to synthesize Mo_2_CT_*x*_. This also indicates that the range of MXene precursors can be further expanded to Ga‐containing carbides and nitrides.

### 2.2 Bottom‐Up Synthesis

**Figure 3:**
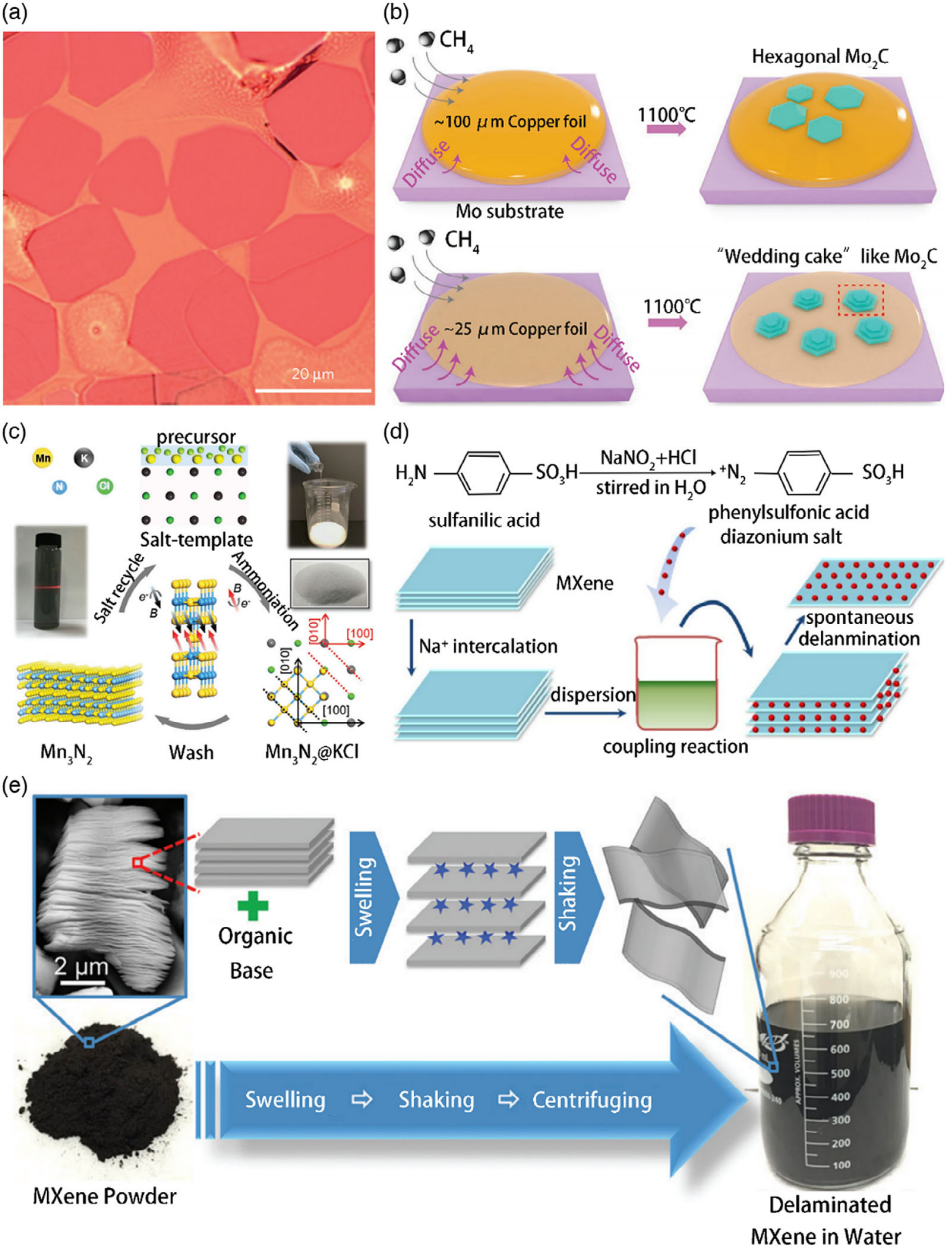
a) Typical optical images of ultrathin α‐Mo_2_C crystals on copper/molybdenum substrates, showing different regular shapes. b) By changing the thickness of the copper diffusion barrier, the schematic diagram shows the regulation of the growth of “wedding cakes” such as hexagonal single crystals and Mo_2_C crystals. c) Schematic diagram of the synthesis of ultrathin Mn_3_N_2_ flakes (labeled 2D Mn_3_N_2_) using the salt template method. d) Schematic diagram of the layering phase of the aryl diazonium salt‐modified multilayer film MXene. e) Schematic diagram of MXene's layering mechanism. The MXene reaction with organic bases leads to a major expansion of the MXene powder multilayer (bottom left in the figure). Then these layers are layered to form a solid colloidal solution by simple hand shaking or gentle ultrasonic treatment in water (right). In the upper left corner, a typical scanning electron microscopy image of the synthesized Ti_3_CNT_*x*_ multilayer MXene is shown. a) Reproduced with permission.^[11b]^ Copyright 2015, Springer Nature. b) Reproduced with permission.^[^ ^29^ ^]^ Copyright 2019, Wiley‐VCH. c) Reproduced with permission.^[^ ^30^ ^]^ Copyright 2019, Wiley‐VCH. d) Reproduced with permission.^[^ ^32^ ^]^ Copyright 2016, Elsevier. e) Reproduced with permission.^[^ ^31^ ^]^ Copyright 2015, Royal Society of Chemistry.

The top‐down synthesis method is widely used for preparing MXenes. However, the chemically derived functionalized MXene sheets prepared using this method are very small (with a lateral size ranging from hundreds of nanometers to 10 μm), suffer from serious structural defects, and are blocked by hydroxyl and/or other oxygen groups. These groups are capped and fluorine is present on their surface. Although MXenes exhibit a complex lattice structure and multielement composition, some researchers have successfully reported the use of bottom‐up methods such as chemical vapor deposition (CVD) to develop MXene materials. Xu et al. developed for the first time, a CVD method using methane as the carbon source and a Cu foil sitting placed on a molybdenum foil substrate at a temperature greater than 1085 °C to create high‐quality 2D ultrathin α‐Mo_2_C crystals with a thickness of a few nanometers.^[11b]^ **Figure** **3**a shows that the ultrathin α‐Mo_2_C crystals grew for 5 min at 1090 °C. Interestingly, all the ultrathin α‐Mo_2_C sheets, including triangles, rectangles, hexagons, octagons, nonagons, and dodecagons, showed a fixed form, providing typical dogmatic crystal characteristics. Most of the crystals had a lateral dimension of ≈10 μm and a thickness of 3 nm. It is well known that α‐Mo_2_C is a standard superconducting TMC in which Mo atoms are arranged in a slightly twisted hexagonal close‐packed arrangement and C atoms are arranged to form an orthorhombic structure in the octahedral voids of the Mo lattice. At the liquid Cu/Mo interface, the higher growth temperature melted Cu, leading to the formation of a Mo–Cu alloy. Subsequently, the Mo atoms diffused from the interface to the surface of liquid Cu and reacted with the carbon atoms generated by the decomposition of methane to form Mo_2_C crystals. Here, the use of extremely low concentrations of methane is the key to obtaining ultrathin α‐Mo_2_C crystals that can replace graphene. Moreover, rapid cooling after the CVD growth is critical to obtain clean ultrathin α‐Mo_2_C crystals without Mo nanoparticles on the surface. Zhao et al. recently found that the atomic structure of Mo_2_C can be regulated between the uniform AA‐stacked T‐phase and the Bernal‐stacked “wedding cake” by adjusting the thickness of the copper diffusion barrier.^[^ ^29^ ^]^ They used an environmental pressure CVD system for the growth of Mo_2_C. A copper foil was mounted on the supporting Mo substrate as the catalyst and diffusion barrier and the resulting assembly was placed in a quartz reactor. The copper foil melted and wetted the entire Mo substrate at temperatures greater than 1100 °C. The rise in the reaction temperature contributed to the formation of the Cu–Mo alloy, and the resulting Cu–Mo alloy was used for surface segregation. To form the Mo_2_C crystals, the separated Mo was further reacted with the carbon precursor. The thickness of the base copper foil affected the growth rate of the Mo_2_C crystal, and the thickness of the base copper foil was affected by the diffusion rate of Mo. The Mo_2_C crystals developed rapidly on the copper surface when the thickness of the copper and aluminum foils was reduced from 100 to 25 μm, creating a “wedding cake” structure, which facilitated the diffusion of molybdenum atoms from the edges to the center (Figure 3b). Recently, a high‐yield salt template method has been developed for synthesizing atomically thin TMNs via the bottom‐up synthesis approach using ammoniating metal oxides. This method provides a facile route to obtain ultrathin sheets using room temperature magnetism. In 2018, Xiao et al. reported the efficient synthesis of ultrathin 2D Mn_3_N_2_ using the salt template method.^[^ ^30^ ^]^ They found that the crystal lattice of KCl fitted well with Mn_3_N_2_, and single‐crystal 2D Mn_3_N_2_ could be grown on the surface of KCl. To the best of our knowledge, Mn_3_N_2_ flakes are the first ultrathin transition metal nitrides with magnetic properties and room temperature solution stability reported to date. Figure 3c shows the schematic for the synthesis of 2D Mn_3_N_2_. First, an ethanol solution of MnCl_2_ was coated on the surface of the KCl salt template and dried to form a thin layer of MnCl_2_ (labeled MnCl_2_@KCl). Then, a clear precursor solution of MnCl_2_ was poured on 200 g KCl, and the MnCl_2_@KCl powder was dried at a constant temperature of 750 °C under ammonia water. During the amination reaction, MnCl_2_@KCl transformed into Mn_3_N_2_@KCl. To obtain 2D Mn_3_N_2_, the transformed KCl salt prototype was washed with deionized water. Finally, 2D Mn_3_N_2_ was well dispersed to form a dark colloidal solution, as confirmed by the Tyndall effect.

### 2.3 Delamination

Like the case of any new material intended for large‐scale applications, material availability and processability have always been the rate‐limiting steps in the evaluation of the potential applications of MXenes. The effectiveness of MXene materials is hampered by the need to overcome the high cohesive van der Waals energy between MXene sheets. To date, most MXene applications are focused on stacking multiple layers of MXenes. To decompose MXene flakes in layers, Gogotsi and Naguib et al. first used dimethyl sulfoxide (DMSO) as an intercalant to form multiple layers of MXene Ti_3_C_2_ and then effectively separated the 2D flakes by ultrasound. Compared with multilayer MXenes, single‐layer MXenes show significantly improved electrochemical performance. Because of the high boiling point of the solvent used, it is difficult to completely remove it, which leads to poor MXene delamination. In addition, some DMSO molecules remain on the surface of MXenes even after rinsing with a large amount of deionized water. These molecules then bond together to increase the thickness of the sheet. In 2015, Naguib et al. reported a general method for the large‐scale layering of MXenes using relatively large organic bases, namely tetrabutylammonium hydroxide (TBAOH) and amine instead of DMSO as an intercalant to delaminate Ti_3_CN, V_2_C, and Nb_2_C.^[^ ^31^ ^]^ These bases reacted with MXenes to produce inserted MXenes with increased d‐spacings (as compared with the original MXenes), that is, enlarged MXenes (Figure 3e). Although this layering method is suitable for developing specific MXene materials, the resulting MXene flakes do not fulfill the high quality requirements of various applications requiring completely layered and large‐sized flakes. Hence, Wang et al. proposed a new method based on diazonium surface chemistry for layering MXenes (Ti_3_C_2_).^[^ ^32^ ^]^ Figure 3d shows the schematic of the layering strategy used by them to embed Na^+^ in multilayer Ti_3_C_2_. After embedding Na^+^ in Ti_3_C_2_, diazonium sulfonate was used for the surface modification of Ti_3_C_2_. The intercalation of metallic Na^+^ ions not only improved the electronic conductivity and electrochemical performance of the multilayer Ti_3_C_2_ MXene, but also weakened its interlayer adhesion and accommodated aryl diazonium salts because the interlayer spacing formed a more open structure.

## 3 Electrical Properties

As discussed earlier, because of their peculiar physical and chemical properties (distinct from those of their bulk forms), 2D materials containing transition metal atoms have been extensively investigated over the past few decades. Computational studies focusing on a broad class of 2D materials and expanding their possible applications have gained immense attention. Due to their great electrical conductivity and bandgap induced by surface termination, the MXene family integrate the properties of both graphene and semiconducting 2D materials. The broad family of MXenes can be classified according to their crystal structure (monometallic, bimetallic solid solution or ordered structure, vacancy‐ordered type). On the one side, MXenes provide excellent electrochemical efficiency with their unusual combination of metal conductivity, hydrophilicity, and high‐charge surface. On the other hand, the semiconducting properties of MXene are determined by proper surface functional groups. In this section, we will discuss the electronic properties of MXene based on the different surface termination.

### 3.1 Surface Termination

**Figure 4:**
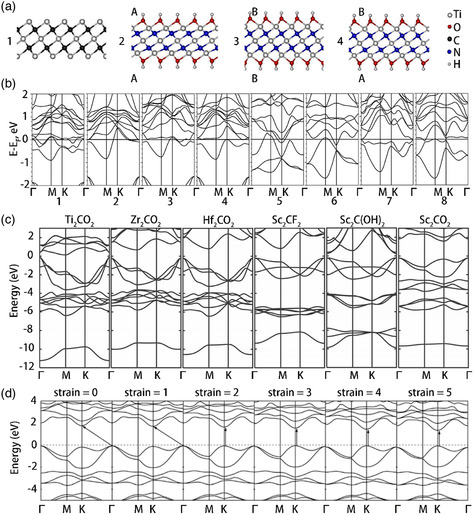
a) Schematic diagram of the atomic structure of the double‐sided surface terminal of MXenes, there are three main configurations: two symmetrical (AA and BB) and asymmetrical configurations AB. b) Near‐Fermi electronic bands for bare MXene Ti_3_C_2_(1) and for its hydroxylated derivatives Ti_3_C_2_(OH)_2_(2–4) with possible configurations of OH covering of external Ti sheets: AA (2), BB (3), AB (4). Ti_3_N_2_(5) and for hydroxylated derivatives Ti_3_N_2_(OH)_2_(6–8) with possible configurations of OH covering: AA (6), BB (7), AB (8). c) The structure of the energy band in the M_2_CT_2_ MXene semiconductor device. The energy of Fermi is zero and the electronic band structure of MXenes is greatly influenced by transition metal elements and surface functional groups. d) Band structure in each state of tensile strain. The Fermi level is set to zero, and the arrow indicates the direction of the bandgap from the VBM state to the coalbed gas state. a,b) Reproduced with permission.^[^ ^33^ ^]^ Copyright 2013, Elsevier. c) Reproduced with permission.^[^ ^34^ ^]^ Copyright 2013, Wiley‐VCH. d) Reproduced with permission.^[^ ^35^ ^]^ Copyright 2014, Royal Society of Chemistry.

Usually, nonterminated MXenes are metallic because of their outer transition metal layer and exhibit a high density of states on the Fermi surface. It should be noted that MXenes are not original carbide (nitride) nanolayers, but they are terminated by various functional groups. The presence of functional groups on the surface of MXenes contributes to the dramatic changes in their properties. In the self‐consistent charge density function tight binding (DFTB) process, all the measurements of the structure and electronic properties of pure and functional MXene layers are performed. Ivanovskii et al. studied the structural and electronic properties of novel MXene compounds—2D pristine carbonitrides Ti_3_C_2−*x*_N_*x*_ and their hydroxylated derivatives Ti_3_C_2‐*x*_N_*x*_(OH)_2_—by carrying out their DFTB calculations.^[^ ^33^ ^]^ The side views of the optimized atomic structures of Ti_3_C_2_ and the Ti_3_N_2_–Ti_3_N_2_(OH)_2_ hydroxylated derivatives are shown in **Figure** **4**a. The possible configurations of the hydroxyl group covering the outer titanium sheet are as follows: A—all the hydroxyl groups were located in the hollow positions between the three adjacent nitrogen atoms and B—all the hydroxyl groups were located on the top of the nitrogen atoms. For the double‐sided surface terminal, three main configurations of Ti_3_N_2_(OH)_2_ were described: two symmetrical configurations (AA and BB) and one asymmetrical configuration (AB). Figure 4b shows the near‐Fermi electron bands of the bare Ti_3_C_2_ and its hydroxylated derivative Ti_3_C_2_(OH)_2_.^[^ ^33^ ^]^ It was found that unlike the case of the binary MXene (Ti_3_C_2_), in the original carbonitrides, carbon was replaced by nitrogen, resulting in a decrease in the lattice parameter. These materials are similar to metals, and their electronic spectrum includes three independent sub‐bands consisting of mixed N2p‐Ti3d states, mixed C2p‐Ti3d states forming the covalent Ti—N and Ti—C bonds, and near‐Fermi Ti3d states.

**Figure 5:**
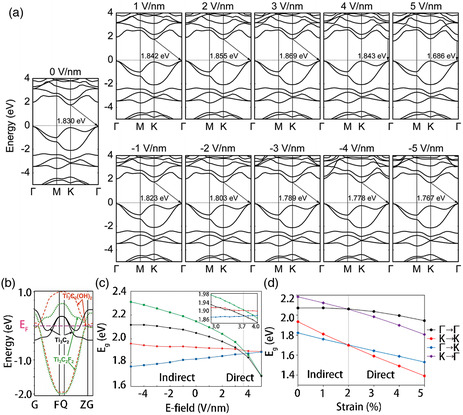
a) Band structure under each perpendicular external E‐field in monolayer Sc_2_CO_2_. The degree of Fermi has been set to zero and the arrows reflect the excited electron–hole pairs’ recombination pathways. b) The energy band structure of single‐layer MXene is calculated, with —OH and —F surface termination and no termination (Ti_3_C_2_), showing the change from metal to semiconductor caused by surface chemical changes. c) As a function of the electrical field, the energy difference varies from the lowest conduction band to the highest valence band. The G to G, K to K, K to G, and n d G to K gaps are represented, respectively, by the black, red, blue, and green lines. d) Shift of energy gaps from the valence band to the conduction band as a function of strain; (black) Γ to Γ, (red) K to K, (blue) Γ to K, and (purple) K to Γ gaps. a,c) Reproduced with permission.^[^ ^35^ ^]^ Copyright 2014, Royal Society of Chemistry. b) Reproduced with permission.^[^ ^24^ ^]^ Copyright 2011, Wiley‐VCH. d) Reproduced with permission.^[^ ^36^ ^]^ Copyright 2014, American Chemical Society.

Kawazoe and coworkers investigated the electronic properties of the most stable structures of MXene‐functionalized frameworks. Although most MXenes are metallic, the results showed that Sc_2_CX_2_ (X = F, OH, and O), Ti_2_CO_2_, Zr_2_CO_2_, and Hf_2_CO_2_ are semiconductors. For Sc_2_X_2_ with X = F, OH, and O, the energy gaps were 1.03, 0.45, and 1.8 eV, respectively. For Ti_2_CO_2_, Zr_2_CO_2_, and Hf_2_CO_2_, the energy gaps were 0.24, 0.88, and 1.0 eV, respectively.^[^ ^34^ ^]^ Thus, it can be concluded from the measured band structure (Figure 4c) that Sc_2_C(OH)_2_ had a direct bandgap, whereas the other semiconductors showed an indirect bandgap. In the periodic table, Ti, Zr, and Hf belong to the same group, and they have the same number of electrons in their shells. Therefore, when the same kind of functionalization is carried out, the devices based on MXenes containing these elements exhibit similar metal‐to‐semiconductor behavior near the Fermi energy. When terminated by a hydroxyl group and a fluorine group, the band structure exhibits semiconductor characteristics, and the separation between the valence band and conduction band is 0.05 and 0.1 eV (**Figure** **5**b), respectively.^[^ ^24^ ^]^ Therefore, it can be concluded that the electronic structure of the peeled maximum layer can be modified by adjusting the functional groups present on its surface.

As reported previously, an indirect‐to‐direct bandgap transformation should be induced in modern 2D optoelectronics and optic materials to realize their practical applications. As the electric field can be accurately controlled using different methods, an external electric field is typically applied to several layered materials to modify their electronic structure. As shown in Figure 5a, the indirect‐to‐direct bandgap transformation of a single layer of Sc_2_CO_2_ was studied using density function theory calculations and applying a vertical external electric field.^[^ ^35^ ^]^ The bandgap decreased rapidly by 183 meV following the indirect‐to‐direct bandgap transformation when the positive external electric field was increased only by 2 V nm^−1^. It should be noted that the monolayer Sc_2_CO_2_ reduced significantly under the action of the external electric field. The modifications are in direct contrast with the findings reported for original monolayer materials. It is well understood that the electronic properties of 2D materials, including the indirect band distance, which is particularly important for these materials, can be modified by applying pressure. The strain applied to single‐layer materials alters the distance between atoms and the relative location of atoms, influencing the electronic structure of the material. This effect can occur easily via lattice mismatch or mechanical loading on the substrate. Therefore, Chung and coworkers studied the effect of strain on the bandgap characteristics of semiconductor‐terminated MXenes used in optical devices (Figure 4d and 5c).^[^ ^35^, ^36^ ^]^ To investigate the effect of strain on the indirect‐to‐direct bandgap transition of Sc_2_CO_2_, they calculated the Γ to Γ and K to K direct bandgaps and the Γ to K and K to Γ indirect bandgaps, which are functions of strain, of a single layer of Sc_2_CO_2_ (Figure 5d).^[^ ^36^ ^]^ Due to these changes, the single‐layer SC_2_Co_2_ underwent an indirect‐to‐direct bandgap transition at a relatively small critical tensile strain of ≈2%. In addition, an increase in the tensile strain from 0% to 5% resulted in a gradual decrease in the bandgap of this system. From these results, it can be concluded that the bandgap of single‐layer MXenes can be modulated by applying electric field and strain to render them suitable for various applications.

### 3.2 Work Function

**Figure 6:**
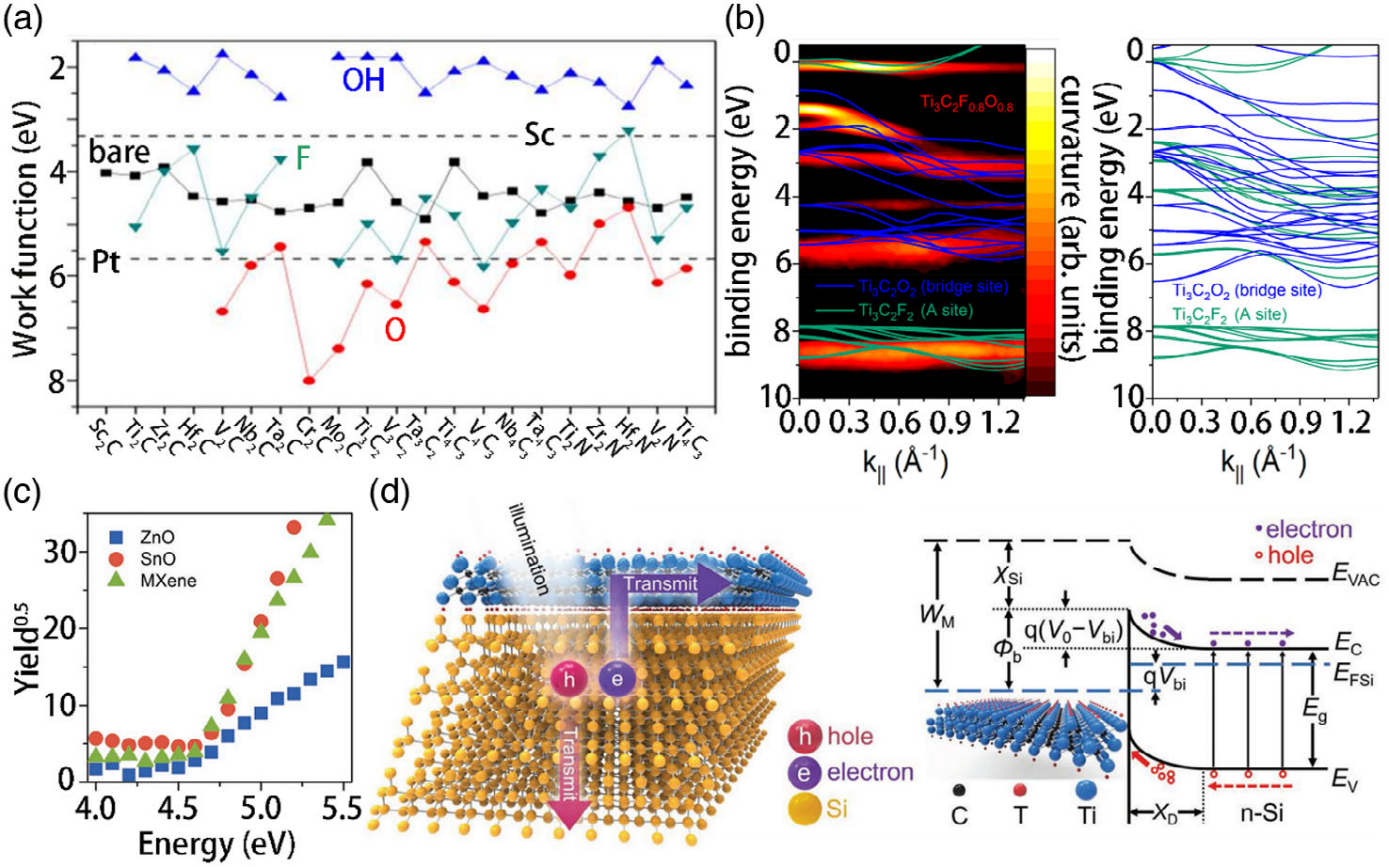
a) Calculated WFs of bare and terminated MXenes in comparison with Sc and Pt. b) On the left is the ARPES curvature spectrum of Ti_3_C_2_F_0.8_O_0.8_ (yellow‐red), including the high symmetry of Ti_3_C_2_F_0.8_O_0.8_ (blue) and Ti_3_C_2_F_2_ (F is only adsorbed on the A site). In the right figure, the complete energy band measurement is given. c) The working feature of the contact material, the n‐type zinc oxide conduction band and the tin oxide valence band were calculated using photoelectron spectroscopy in the air (PESA) technology. d) Schematic diagram of Ti_3_C_2_T_*x*_/n‐Si heterostructure and its electronic band diagram. a) Reproduced with permission.^[^ ^37^ ^]^ Copyright 2016, American Chemical Society. b) Reproduced with permission.^[^ ^38^ ^]^ Copyright 2019, American Chemical Society. c) Reproduced with permission.^[^ ^39^ ^]^ Copyright 2018, Wiley‐VCH. d) Reproduced with permission.^[^ ^40^ ^]^ Copyright 2017, Wiley‐VCH.

MXenes exhibit excellent electrical conductivity, and hence are applied in electrochemical systems as electrodes. The electron WF of MXenes is the difference in the energies of their Fermi and vacuum levels and describes the minimum energy needed to remove electrons from their surface. The change in the WF of bare MXenes can be correlated with the induced dipole of the surface caused by the functional groups and the shift of the material at the Fermi level caused by the redistribution of electrons. Here, we discuss the potential of MXenes for application in 2D semiconductors as Schottky barrier‐free metal contacts. The WF of metal MXenes, as shown in **Figure** **6**a, varies from 1.8 to 8 eV.^[^ ^37^ ^]^ The WF of MXenes is temperature sensitive. Compared with the bare surface, O‐termination increases the WF, whereas the OH group reduces the WF of MXenes. F‐termination shows a mixed effect on the WF of MXenes depending on the particular material. Interestingly, all hydroxyl‐terminated MXenes exhibit relatively low WF, even lower than that of Sc metal. Some O‐terminated MXenes show relatively high WF, much greater than that of Pt, which exhibits the highest WF among metals. F‐terminated MXenes show a WF (W_F_) intermediate to those of O‐ (W_O_) and OH‐terminated (W_OH_) MXenes. Furthermore, *W*_F_ and *W*_O_ show a positive correlation, whereas a negative correlation is observed between *W*_OH_ and *W*_O_. The change in the WF of MXenes after F‐functionalization can be attributed to the change in their surface dipole moment induced by the functionalization. The hydroxyl end often leads to a negative (positive) dipole moment of the surface, thus reducing (increasing) the WF, and fluorine may induce a negative or positive dipole moment on the surface depending on the MXene material. Therefore, modulating the surface functional groups of hydroxyl‐terminated single photonic crystals is necessary to achieve an ultralow (or ultrahigh) WF. Recently, Schultz et al. calculated the WF of Ti_3_C_2_T_*x*_ at different annealing temperatures, and found that the WF of Ti_3_C_2_T_*x*_ varied from 3.9 to 4.8 eV depending on its surface composition (Figure 6b).^[^ ^38^ ^]^ The WF of Ti_3_C_2_T_*x*_ increased from 3.9 to 4.8 eV upon vacuum heating, most likely because of water desorption, carbon‐dominated contamination, and the presence of OH species. The WF then decreased to 4.1 eV at higher temperatures because of fluorine desorption. However, another study demonstrated for the first time about the application of large‐area MXenes (Ti_3_C_2_) as electrical contacts (gate, source, and drain) for producing n‐type and p‐type oxide thin‐film transistors (TFTs). This could be realized because the WF of Ti_3_C_2_ fits well with the band edges of zinc oxide (ZnO) and tin oxide (SnO), forming good Ohmic contacts. The WF (*φ*) of MXenes (Ti_3_C_2_) is very important because they can be used as contact materials for n‐type (ZnO channel) and p‐type (SnO channel) transistors. Airborne photoelectron spectroscopy was used to calculate the WF of contact materials (MXene), the n‐type ZnO conduction band (*E*_C_, or electron affinity), and the SnO valence band (*E*_V_, or ionization energy), as shown in Figure 6c.^[^ ^39^ ^]^ The measured MXene, *E*_C_, and *E*_V_ values were 4.60, 4.58, and 4.65 eV, respectively. These values indicate that as the *φ* of n‐type (p‐type) semiconductors is deeper (shallower) than the *E*_C_ (*E*_V_) standard, the potential barrier for carrier injection into the respective transistor channel is negligible. In addition, Schottky contacts with silicon nitride can be formed by Ti_3_C_2_T_*X*_ with a WF of 4.37 eV, which acts as a transparent electrode and creates a strong built‐in electric field in n‐Si near the interface between Ti_3_C_2_T_*X*_ and n‐Si. The photon‐induced carriers produced in the semiconductor are separated by the built‐in electric field when the Ti_3_C_2_T_*X*_/n‐Si heterostructure is exposed to light. These photon‐induced carriers then recombine through an external circuit to produce an electrical signal. Figure 6d shows the band diagram of the nonequilibrium Schottky junction of the Ti_3_C_2_T_*X*_/n‐Si heterostructure under illumination.^[^ ^40^ ^]^ The electron–holes excited in or near the space charge area were separated by *V*_o_, the holes were transported to Ti_3_C_2_T_*X*_, and the electrons were transported to the metal back electrode through n‐Si. Thus, the MXene film showed potential for different electronic applications as touch‐sensitive devices. These results will help to further explore novel 2D materials.

## 4 Applications

Memory for storing information in modern information technology in the field of science and technology has significantly improved human lifestyle. Depending on the data retention capability, memories can be divided into volatile and nonvolatile memories. Nonvolatile memory is essential for the next generation of digital technology. Currently, the most studied MXene‐based nonvolatile memory can be classified into FET‐based flash memory and resistive random access memory (RRAM). Specifically, structure of FET‐based flash memory is similar to that of FET, except that there is an additional charge storage layer inserting between the gate dielectric and the semiconductor layer. Carriers may switch from the semiconducting channel to the floating gate through quantum tunneling or thermal radiation when a proper programming voltage is applied. As the charge trapped in the floating gate partially shields the electric field between the control gate and the semiconductor which induces a shift in the threshold voltage of the transistor. The high integrity, multibit data storage and nondestructive reading make flash memory as promising candidate for high‐density data storage. Another type of nonvolatile memory that has been widely studied is RRAM, which is composed of a resistance switchable material sandwiched between two electrodes. The information storage of RRAM is based on the physical bistability of the switching material, which stems from the change in the intrinsic properties of the material. The mechanisms of resistance switching include space charge and traps, filament conduction, charge transfer, conformal change, tunneling effect, and ion conduction. In this section, the exploration of MXene‐based memory devices will be discussed.

### 4.1 MXene‐based Transistors

Since the successful exfoliation of one atom‐thick graphene from highly oriented pyrolytic graphite, various 2D materials have been prepared in the past decade.^[^ ^41^ ^]^ Due to its extremely high carrier mobility, graphene has gained worldwide attention; however, its gapless band structure severely limits its practical application in FETs.^[^ ^42^ ^]^ Therefore, the use of TMDs as channel materials for FETs has recently been proposed because they exhibit a suitable energy bandgap and reasonable carrier mobility. Schottky junction contacts are formed between the source/drain electrodes and the channel in TMD‐based FETs; thus, the barrier height at the junction interface has a significant effect on the polarity and efficiency of this system. MXenes are a new family of 2D materials that exhibit hydrophilic surfaces and high conductivity. Since their discovery in 2011, MXenes have proven to be promising candidates for energy storage applications. Due to their intrinsic 2D structure with high density of states, and high WF, MXenes with large contact surfaces exhibit huge potential for application as floating gates in memory devices. The system manufacturing process can be greatly simplified by this feature, which would otherwise involve complex processes (e.g., CVD, epitaxial growth, or mechanical lift‐off). These methods have been used in FET devices based on other materials, such as carbon nanotubes, nanowires, or graphene. Duan et al. reported a vertically stacked graphene MoS_2_ FET for the first time, and found that it exhibited a high current density and high switching ratio when graphene was used as the adjustable source electrode. Graphene electrodes have been extensively investigated since this discovery and have shown better electrical properties than other metal electrodes. Lee et al. also fabricated a versatile and transparent FET using molybdenum disulfide with graphene as the gate electrode and boron nitride as the dielectric layer, allowing the integration of the FET and 2D electronic components.

**Figure 7:**
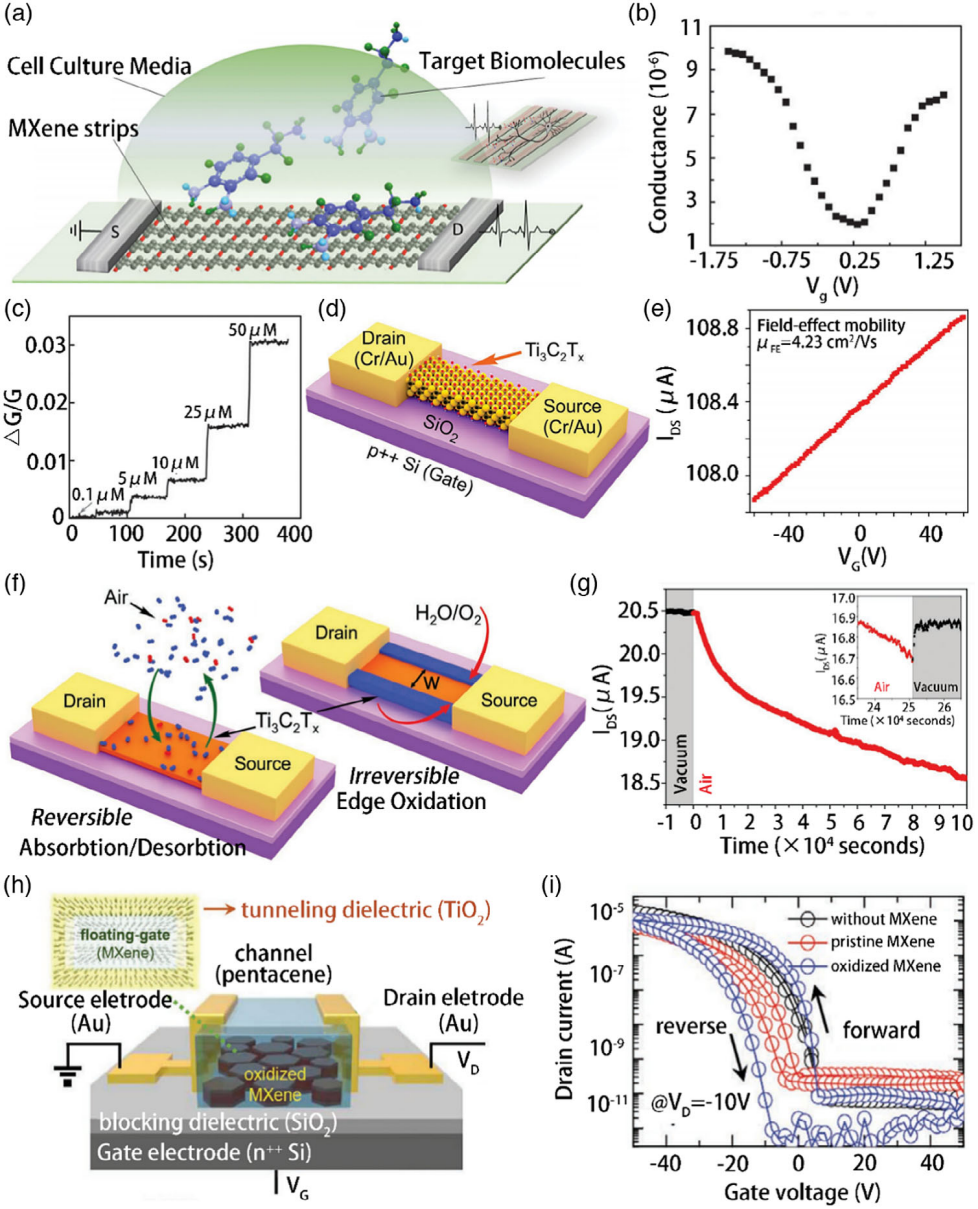
a) Schematic diagram of MXene field effect tube biosensor. b) The device's transfer curve exhibits bipolar characteristics and c) its response to dopamine concentrations. d) Schematic of a Ti_3_C_2_T_*x*_‐based FET. e) *I*_DS_–*V*_G_ dependence for the device, as shown in (d). f) Schematic diagram of the phenomenon that decreases the conductivity of airborne Ti_3_C_2_T_*x*_ devices. g) Representative device detection of intrusion‐time dependency of air‐exposed Ti_3_C_2_T_*x*_ FETs. The unit is first held in a vacuum (black dot) and then in the air (red dot). h) Schematic illustration of MXene NFGTMs which use MXene as floating gate. i) Transfer characteristics of MXene‐based transistors with different hysteresis behaviors. a–c) Reproduced with permission.^[22a]^ Copyright 2016, Wiley‐VCH. d–g) Reproduced with permission.^[^ ^43^ ^]^ Copyright 2016, Wiley‐VCH. h,i) Reproduced with permission.^[21a]^ Copyright 2020, Wiley‐VCH.

In 2015, Shi and coworkers developed a simple and effective FET manufacturing technology based on ultrathin conductive T_3_C_2_‐MXene micropatterns (**Figure** **7**a), which is used for highly sensitive and label‐free detection of dopamine and monitoring the hippocampal neuron spike activity.^[22a]^ To the best of our knowledge, this is the first demonstration of the detection of biological events using MXenes in a cell model. The grid voltage was applied through a silver/silver chloride electrode submerged in the plating solution. As shown in Figure 7b, the conductance of the system could be modulated by applying different gate voltages, and V‐shaped bipolar field effect characteristics could be observed. The amount of pulsed dopamine released into the biosensing chamber was precisely controlled using a femtoliter‐resolved microinjector. In addition, the FET operated in the p‐channel mode. The change in the conductance of the FET system could be calculated in real time to represent the binding event when dopamine molecules were released to interact with the MXene surface (Figure 7c). Therefore, this simple method of manufacturing MXene FET biosensors is highly relevant for a wide range of biological and medical applications. The potential of MXenes for application in electronic devices, such as transistors and sensors, has not been completely realized yet. Sinitskii and coworkers prepared a FET centered on a single‐layer Ti_3_C_2_T_*x*_ sheet and investigated its electronic properties.^[^ ^43^ ^]^ The transistor exhibited a high conductivity of 4600 ± 1100 S cm^−1^ and a field‐effect electron mobility of 2.6 ± 0.7 cm^2^ V^−1^ s^−1^ (Figure 7d). The external field applied through the gate electrode changed the Fermi level of the MXene sheet, thereby changing the *I*_DS_ of the transistor. Figure 7e shows that the *I*_DS_ of the transistor increased with an increase in its *V*_G_, indicating that electrons were the main charge carriers in the operation of this transistor. The Ti_3_C_2_T_*x*_ field‐effect electron mobility (μFE) of the transistor was estimated by fitting its *I*_DS_–*V*_G_ curve. Finally, the environmental stability of the Ti_3_C_2_T_*x*_ flakes in humid air was evaluated using them as FETs. As shown in Figure 7g, the drain–source current decreased linearly with time after the initial exponential decay. Even after exposure to air for 70 h, the Ti_3_C_2_T_*x*_ flakes were very stable and retained high conductivity. Recently, it has been reported that MXene–titania core–shell nanosheets exhibit good hole‐trapping capabilities. Cho and coworkers synthesized a series of MXene–TiO_2_ core–shell nanosheets by controlling the surface oxidation of the MXene to prepare NFGTM (Figure 7h).^[21a]^ The storage performance was optimized by adjusting the thickness of the oxide layer formed on the surface of the MXene. The prepared MXene NFGTMs exhibited excellent nonvolatile storage characteristics, including a large storage window (>35.2 V), high program/erase current ratio (≈10^6^), low off current (<1 pA), long retention time (>10^4^ s), and cycle durability (300 cycles). Figure 7i shows the transfer characteristics (drain current vs gate voltage) of the transistor emphasizing the role of the MXene floating gate. The device without the oxide layer showed slightly larger storage window (4.2 V) than that with the oxide layer. This can be attributed to the trapping sites present in the MXene. After the oxidation, a titanium dioxide layer was formed on the surface of the MXene, causing the storage window to increase to 20.6 V.

**Figure 8:**
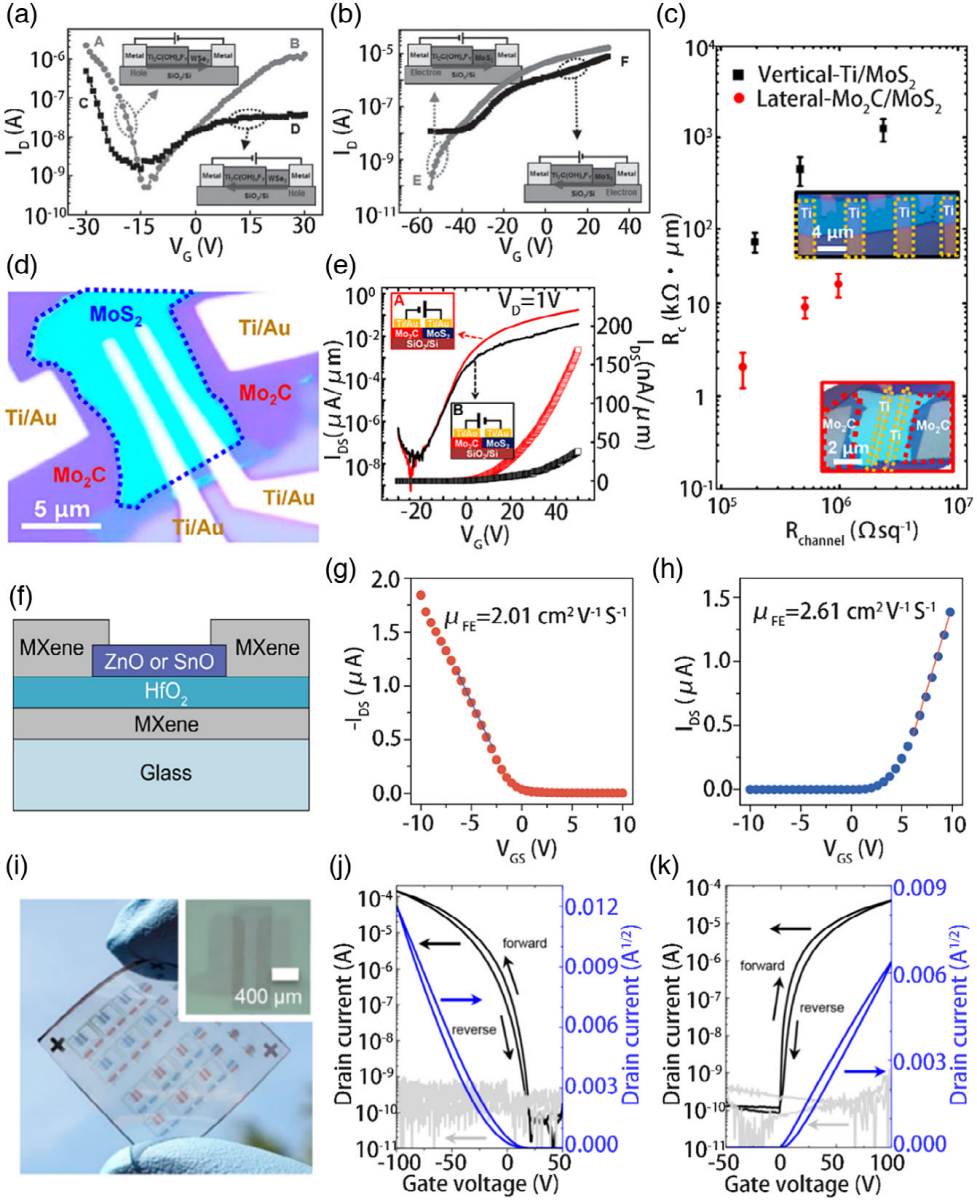
a,b) Transfer curves of a) Ti_2_CT_*x*_/WSe_2_ FET, and b) Ti_2_CT_*x*_/MoS_2_ FET. c) *R*_c_ values calculated with various channel sheet resistance (*R*_channel_) values obtained through gate modulation using the four‐probe measurement method for vertical Ti/MoS_2_ contact (black rectangles) and lateral Mo_2_C/MoS_2_ contact (red circles). d) An optical image of the MoS_2_/Mo_2_C lateral heterostructure FET device and e) its transfer curve. f) Schematic structure of the TFTs with all‐MXene (Ti_3_C_2_) contacts. The mobility extraction for the g) P‐type TFT and the h) N‐type TFT. i) Photographic image of MXene‐electrode‐based OFETs, inset is an optical microscopy image of OFET. Transfer characteristics on pure Ti_3_C_2_T_*x*_ electrodes of j) p‐type pentacene and k) n‐type PTCDI‐C_8_OFETs with NH_3_‐doped MXene electrodes field effect transistors. a,b) Reproduced with permission.^[^ ^44^ ^]^ Copyright 2016, Wiley‐VCH. c–e) Reproduced with permission.^[^ ^45^ ^]^ Copyright 2018, American Chemical Society. f–h) Reproduced with permission.^[^ ^39^ ^]^ Copyright 2018, Wiley‐VCH. i–k) Reproduced with permission.^[^ ^46^ ^]^ Copyright 2019, American Chemical Society.

MXenes are also promising candidates for 2D FET electrodes. However, only a few reports are available on the use of MXenes as 2D electrodes. Sungjoo et al. prepared WSe_2_ and MoS_2_ FETs with Ti_2_C(OH)_*x*_F_*y*_ electrodes to extend the use of MXene materials in 2D electronic devices. Ti_2_C(OH)_*x*_F_*y*_ shows higher WF (≈4.98 eV) than graphene (4.6 eV) and lower Schottky barrier than WSe_2_ and MoS_2_, indicating that it is a promising substrate for semiconductor inverter 2D electrodes (complementary metal oxide semiconductor, CMOS). To analyze the electronic properties of Ti_2_C(OH)_*x*_F_*y*_, the transport characteristics of WSe_2_/Ti_2_C(OH)_*x*_F_*y*_ and MoS_2_/Ti_2_C(OH)_*x*_F_*y*_ FETs were investigated. The inner diameter and thickness features of these transistors measured at room temperature are shown in **Figure** **8**a,b, respectively.^[^ ^44^ ^]^ After the analysis, when the Cr/Au electrode electrons were injected into WSe_2_, the measured current was higher than the positive *V*_DS_ current, where the electrons were supplied by Ti_2_C(OH)_*x*_F_*y*_. Positive *V*_GS_ was applied in both cases, so the electron conduction was expected to dominate the WSe_2_ path. However, when a negative *V*_GS_ was applied, a better hole conduction path was formed. Under these conditions, hole carriers were injected from Ti_2_C(OH)_*x*_F_*y*_ into Cr/Au. Figure 8b shows the transfer characteristics of the MoS_2_/Ti_2_C(OH)_*x*_F_*y*_ FETs, where Ti_2_C(OH)_*x*_F_*y*_ is the source electrode and Cr/Pd is the drain electrode. Ti_2_C(OH)_*x*_F_*y*_ showed high power, and a poor Fermi‐level pinning effect was observed at the metal WSe_2_ interface indicating that the use of Ti_2_C(OH)_*x*_F_*y*_ contributed to the lowering of the hole barrier. MXenes with MoS_2_ can form a metal/semiconductor (Mo_2_C/MoS_2_) junction. For potential system applications, the junction has an atomic‐level sharp interface and low contact resistance (1.2 kΩ μm), which may be a key hybrid structural component of the transistors. An optical microscopy image of a FET system manufactured using a lateral Mo_2_C/MoS_2_ junction under partial switching conditions is shown in Figure 8d.^[^ ^45^ ^]^ To investigate its electrical performance, the *I*_d_–*V*_G_ transfer curves of the MoS_2_ FET (*L* = 2.5 μm, *W* = 10.2 μm) were obtained, as shown in Figure 8e. The red curve obtained under a positive *V*_DS_ represents the electrons injected into the Mo_2_C channel from the Mo_2_C electrode, whereas the black curve was obtained under negative *V*_DS_ because of the injection of electrons from the titanium electrode into Mo_2_C. Mo_2_C/MoS_2_ heterostructure FET devices show similar n‐type transport characteristics under positive and negative *V*_DS_. Figure 8c shows the *R*_c_ values calculated from the sheet resistance (*R*_channel_) values of the various channels obtained by gate modulation. An exceptionally low *R*_c_ value was obtained from the lateral Mo_2_C/MoS_2_ junction (20–1.2 kΩ μm, *R*_channel_ 10^6^–10^5^ Ω sq^−1^). This value was approximately two orders of magnitude lesser than the value obtained from the vertical Ti/MoS_2_ junction. The low *R*_c_ value is comparable with the value recorded for 1T‐MoS_2_/2H‐MoS_2_ junctions. This indicates that the lateral contact geometry of the Mo_2_C/MoS_2_ junction formed in this study was consistent with that reported previously for 1T‐MoS_2_. For the contact lengths shorter than the transfer length, *R*_c_ increases at the vertical junction.

Full MXene electrical contacts including gate, source, and drain have been verified in n‐type zinc oxide and p‐type tin oxide TFTs. Alshareef et al. demonstrated for the first time that n‐type and p‐type oxide TFTs can be fabricated with large‐area MXene (Ti_3_C_2_) electrical contacts (gate, source, and drain). The secret to this outcome is that the properties of MXenes (Ti_3_C_2_) fit well with the ZnO and SnO band edges, forming strong Ohmic contacts. Spraying is used to shape MXene films because it is a cost‐effective thin‐film electronics technology compatible with large‐scale processing techniques, such as inkjet printing and roll‐to‐roll processing.^[^ ^39^ ^]^ Figure 8f shows the schematic of the TFT device, which had a typical bottom‐gate staggered structure. The on–off ratios (*I*_on_/*I*_off_) of the p‐type and n‐type TFTs were 1.1 × 10^3^ and 3.6 × 10^6^, respectively. The subthreshold swings (SSs) of both the TFTs were determined from the inverse of the maximum slope of their logarithmic‐scale transfer curves. The SS values of the p‐type and n‐type TFTs were 2.51 and 0.23 V dec^−1^, respectively. The field‐effect mobility of the p‐type and n‐type TFTs were determined from their transfer curves, as shown in Figure 8g,h, respectively. The manufacture of large‐scale and uniform MXene electrode arrays on flexible plastic substrates for use in high‐performance FETs was later demonstrated by Cho and coworkers (Figure 8i).^[^ ^46^ ^]^ The transfer curves of the MXene electrode and the OFET with the MXene and NMX electrodes are shown in Figure 8j,k, respectively. The proposed method is expected to widen the application range of MXenes to other electronic devices based on OFETs, such as organic light‐emitting displays and electronic skins.

### 4.2 MXene‐Based RRAM

**Figure 9:**
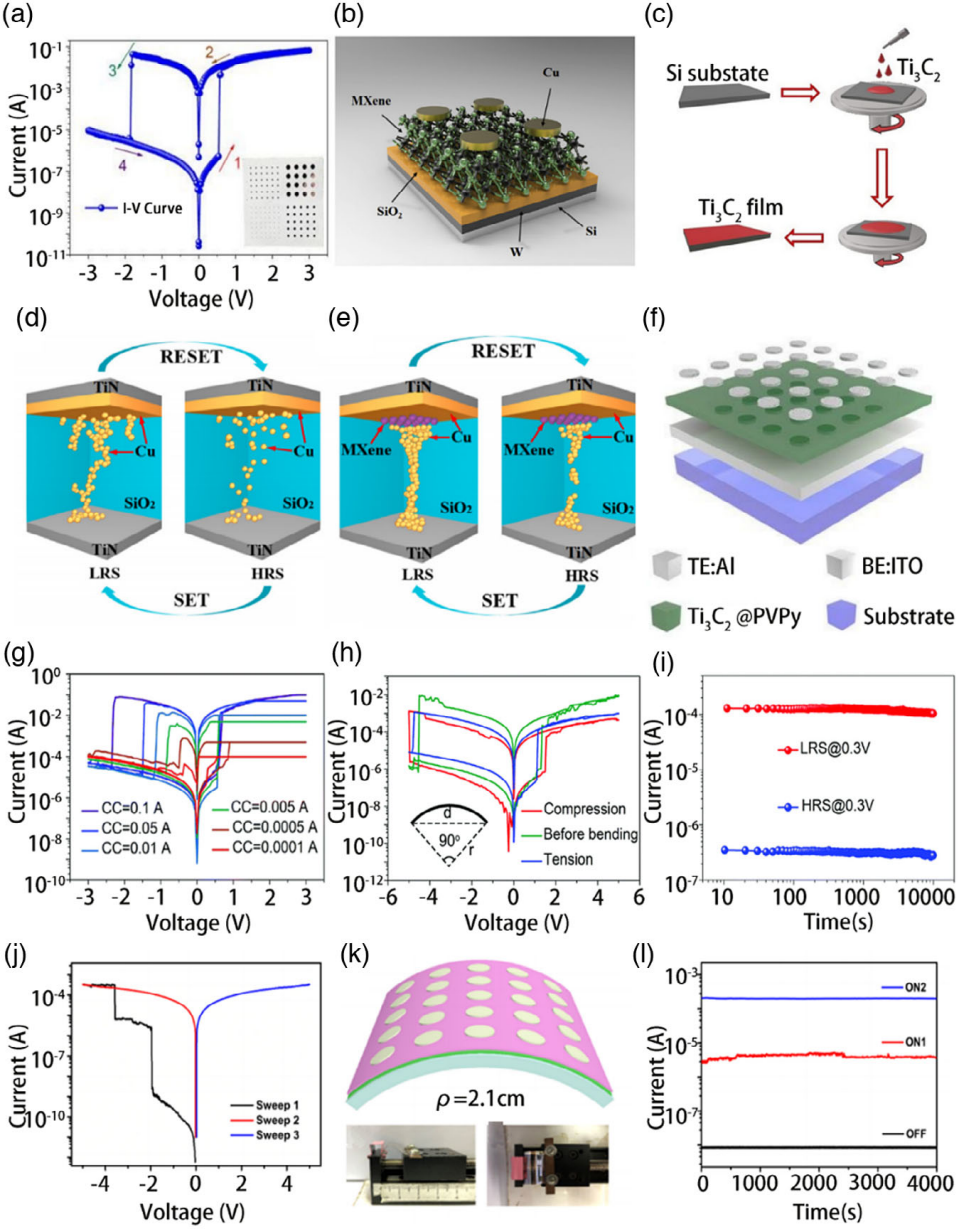
a) Typical *I*–*V* curve of RRAM device: the “1” process indicates that under the forward voltage applied, the device changes to LRS, “3” process represents the transition from LRS to HRS. b) Structure schematic of Cu/Ti_3_C_2_/SiO_2_/W memristive device. c) The process of making Ti_3_C_2_ thin film by spin coating. d) Schematic diagram of the CFs of TiN/Cu/MXene/SiO_2_/TiN and e) TiN/Cu/SiO_2_/TiN devices. f) Structure of Al/Ti_3_C_2_ @ PVPy/ITO device. g) The *I*–*V* curves of the Ti_3_C_2_‐based RRAM device under different *I*_CC_ values. h) The *I*–*V* curves of the flexible RRAM with bending (inset: the condition of bending test: *r* = 13 mm and *d* = 18 mm). i) The retention capability of the Ti_3_C_2_‐based flexible RRAM. j) *I*–*V* characteristic curve of Al/Ti_3_C_2_T_*x*_‐OP MXene/ITO device. k) Schematic diagram of the flexible device based on Ti_3_C_2_T_*x*_‐OP MXene (inset: the front view and the top view). l) Three resistance states after 5000 bending tests. a) Reproduced with permission.^[^ ^50^ ^]^ Copyright 2019, Royal Society of Chemistry. b,c) Reproduced with permission.^[^ ^52^ ^]^ Copyright 2019, Elsevier. d,e) Reproduced with permission.^[^ ^53^ ^]^ Copyright 2020, Elsevier. f–i) Reproduced with permission.^[^ ^50^ ^]^ Copyright 2019, Royal Society of Chemistry. j–l) Reproduced with permission.^[21b]^ Copyright 2020, American Chemical Society.

In traditional electronic computers, electronic devices based on the Von Neumann structure perform data calculation and data storage separately, resulting long‐term delay and high power consumption, which limit the applications of traditional memory.^[^ ^47^ ^]^ With the explosive growth of digital information, the requirement for high‐density storage has brought storage devices to the limit of scaling, which presents the most serious problem in following the Moore's Law. Therefore, to improve the performance and storage density of computers, it is imperative to make breakthroughs and innovations in terms of system structure and size reduction.^[^ ^48^ ^]^ As a new type of nonvolatile memory, RRAM has many advantages over traditional memory due to its simple device structure (metal–insulator–metal, MIM), CMOS compatibility, 3D stackability, low power consumption, and high read and write speed. It has gained immense attention and is considered as the most promising candidate for next‐generation nonvolatile storage applications.^[^ ^49^ ^]^ RRAM generally switches to a low‐resistance state (LRS) of the memory using a sweeping voltage and can make the device return to a high‐resistance state (HRS) by applying a reverse voltage (**Figure** **9**a).^[^ ^50^ ^]^ In this process, a larger on/off window indicates the possibility of multilevel storage. Moreover, in the MIM structure, the selection of the resistive switching (RS) layer material is crucial for the operating voltage and stability of the RRAM.^[^ ^51^ ^]^ This is because the RS layer material directly affects the formation and fracture of the conductive filaments (CFs). Most of the metal oxides that have been reported to date as the intermediate layer of RRAM usually require large set/reset voltages. In addition, considering that RRAM devices are often accompanied by various challenges such as biocompatibility, stretchability, stability, and uniformity during the manufacturing and application processes, the development of novel materials is essential to overcome these challenges.

In recent years, the combination of 2D materials and RRAM has made a breakthrough in the application and performance of devices. Due to their unique electrical properties and flexibility, 2D materials have been used to fabricate mainstream memory devices such as flash memory and RRAM. Among the various 2D materials investigated to date for memory applications, MXenes have gained significant scientific and industrial attention due to their excellent metal conductivity and abundant surface functional groups. There have been reports on the use of MXenes for reducing the energy consumption and improving the stability of RRAM devices and achieving bending and stretchable functions. In this section, we compare the electrical performance of recently developed RRAM based on several 2D materials (**Table** **3**). We also discuss the performance and application potential of RRAM based on MXenes.

**Table 3:** Performance of memristors based on 2D materials (including MXenes)

2D‐Materials	Device structure	Set/Reset Voltage	ON/OFF ratio	Cycle number	Retention time	Ref
MoS_2_	Ag/MoS_2_@PVA/Ag	3/−3 V	1.28 × 10^2^	10^3^	10^5^ s	[81]
MoS_2_	Al/2H‐MoS_2_@PVP/ITO	0.7/−0.75 V	10	–	–	[82]
MoS_2_	Al/1T@2H‐MoS_2_@PVP/ITO	1.24 V/–	2 × 10^2^	10^4^	6 × 10^2^ s	[82]
MoS_2_	Al/MoS_2_@PCBM/ITO	2 V/–	3 × 10^2^	–	10^4^ s	[83]
h‐BN	Graphene/h‐BN/graphene	2.7/−0.5 V	10^3^	35	–	[84]
h‐BN	Ag/h‐BN/Au	−1.3 V/1.5 V	1 × 10^2^	1.5 × 10^2^	–	[85]
h‐BN	Ag/h‐BN/Cu foil	0.72/−0.37 V	10^2^	5.5 × 10^2^	3 × 10^3^ s	[86]
h‐BN	ITO/h‐BN/graphene	0.66/−0.9 V	4.8 × 10^2^	5 × 10^2^	5 × 10^4^ s	[87]
WS_2_	Ag/WS_2_/Ag	2.3/−2.3 V	10^3^	1.5 × 10^3^	10^5^ s	[88]
WS_2_	Al/WS_2_/Pt/Ti/SiO_2_/Si	1.62/−1.45 V	10^3^	10^4^	25 h	[89]
MoSe_2_	Au/BaTiO_3_/MoSe_2_/SiO_2_/Si/Al	0.6/−0.6 V	10^2^	10^2^	6 × 10^3^ s	[90]
graphene oxide (GO)	Al/GO/LGAC/Ni nanocrystals/Si	0.6/−0.5 V	10^3^	10^3^	10^5^ s	[91]
MXene (Ti_3_C_2_)	Cu/Ti_3_C_2_/SiO_2_/W	1.7/−0.9 V	10^2^	–	10^3^ s	[92]
MXene (Ti_3_C_2_)	TiN/Cu/MXene/SiO_2_/TiN	0.9/−2 V	10	10^2^	3.5 × 10^3^ s	[93]
MXene (Ti_3_C_2_)	Al/Ti_3_C_2_@PVPy/ITO	0.5/−1.8 V	10^4^	10^4^	5 × 10^4^ s	[50]
MXene (Ti_3_C_2_)	Al/Ti_3_C_2_T_*x*_‐OP MXene layer/ITO	−2.1/−3.7 V/–	10^2.7^:10^4.1^	5 × 10^3^	4 × 10^3^ s	[21b]
MXene (Ti_3_C_2_)	Al/Ti_3_C_2_T_*x*_/Pt	4.8/−3.2 V	–	3 × 10^2^	10^6^	[21c]

MXenes (Ti_3_C_2_) were not immediately used in memristors after being successfully synthesized by Naguib et al. in 2011. One reason is that the mechanical properties and electrical conductivity of MXenes required further investigation. Moreover, the working mechanism of memristors was not clearly elucidated at that time. Recently, there have been reports on the use of MXenes as an insert layer or a functional layer in RRAM. These memristive devices based on MXenes not only presented different MXene film preparation methods, but also led to the investigation of the effect of MXenes on the mechanism and performance of RRAM. Researchers have realized flexible devices and multilevel storage through the application of MXene in memristors.

According to some reports, when used as the RS layer in RRAM, SiO_2_ offers low power consumption and multiple resistance states. In addition, a physical model based on the formation and fracture of CFs has been applied to silica‐based memristors, and it can be called stability, which definitely needs further improvement. To solve this problem, Tong and coworkers reported a nonvolatile bipolar memristor with the Gu/MXene/SiO_2_/W/Si structure, in 2019.^[^ ^52^ ^]^ This was the first report on the use of an MXene film (due to its excellent conductivity) in RRAM to reduce the randomness of its set/reset voltage (Figure 9b). In this study, Ti_3_C_2_ powder was prepared by mixing lithium fluoride salt (LiF) and hydrochloric acid (HCl) with Ti_3_AlC_2_. The Ti_3_C_2_ powder was then dissolved in DMSO to obtain a Ti_3_C_2_ solution with a concentration of 10% (0.3 g mL^−1^). Subsequently, a symmetrical and high‐quality film was obtained through the spin‐coating process (speed: 750 rpm) (Figure 9c). The results showed that the introduction of the MXene restricted the growth area of the CFs and improved their systematic distribution. Therefore, the device exhibited better performance than the memristor with the Cu/SiO_2_/W structure while maintaining low operating voltage robustness (Figure 9d,e).^[^ ^53^ ^]^ It is found that MXene has a profound impact on the wide range of application prospects due to its mechanical properties, stability, and good electrical conductivity. Under voltage excitation, the HRS and LRS mechanisms were space‐charge‐limited conduction and Ohmic behavior, respectively, which are consistent with most reports on memristors. High‐density and high‐reliability RRAMs provide the possibility for the commercialization of memristors and are also the cornerstone in the development of artificial synapses.

In addition to their excellent electrical conductivity, MXenes have abundant surface functional groups. Therefore, the application range of MXenes can be broadened through surface modification engineering. Recently, various MXene composite materials have been synthesized by surface modification. In addition, nonvolatile information memory such as write once read many times (WORMs) and RRAM with huge potential for industrial applications has been developed. As known, stability is critical to the device. MXene is prone to oxidative degradation in the natural environment, which limits the development of MXene materials. Combining MXene with other materials can effectively improve the environmental stability of MXene and further broaden its practical application range. Among them, polymers have the advantages of simple preparation, low price and adjustable functions. Combining polymers with MXene to prepare composite films has become one of the current research hotspots. In 2019, Ding et al. demonstrated the existence of RS in flexible MXene (Ti_3_C_2_)‐polyvinylpyrrolidone (PVPy) composite films.^[^ ^50^ ^]^ The authors compared the performances of a flexible RRAM array with the Al/Ti_3_C_2_ @ PVPy/polyethylene naphthalate structure and a rigid device with the Al/Ti_3_C_2_ @ PVPy/indium tin oxide (ITO) structure (Figure 9f) and found that both devices exhibited bipolar nonvolatile characteristics with a low set voltage (<0.5 V). The Ti_3_C_2_ @PVPy‐based device showed an on/off ratio of up to 10^4^, and exhibited multilevel data storage with the retention time of multilevel information bits exceeding 5 × 10^3^ s when different compliance currents (*I*_cc_) were modulated for the device (Figure 9g). The authors also investigated the electrical performance and retention capacity of flexible devices under bending and stretching conditions (Figure 9h,i). As previously described, the insertion of polymers will reduce the restacking of MXene flakes and improve stability by preventing them from contacting with oxygen. The results suggested that the RRAM devices based on Ti_3_C_2_@PVPy films showed outstanding flexibility against mechanical stress and strain, providing a new direction for the application of flexible electronic devices in biomedicine and electronic skin.

As a widely reported effective modifier, octylphosphonic acid has been utilized to modify Ti_3_C_2_T_*x*_ nanosheets through hydrogen bonding such that phosphorus is uniformly distributed on the monolayer Ti_3_C_2_T_*x*_ (Ti_3_C_2_T_*x*_‐OP) nanosheets. The metal oxide surface treated with phosphonic acid ensures a good control of its spatial redistribution, which greatly promotes the stability of MXene. Based on this observation, Sun et al. used Ti_3_C_2_T_*x*_ modified by octylphosphonic acid (Ti_3_C_2_T_*x*_‐OP) as the active layer for the memory devices with the Al/Ti_3_C_2_T_*x*_‐OP MXene/ITO structure in 2020.^[21b]^ The device exhibited typical WORM characteristics and multilevel storage characteristics. As shown in Figure 9j, the resistance state changed twice at 2.1 and 3.7 V when a sweeping voltage of 0–5 V was applied to the device, leading to the on/off ratios of 10^2.7^ and 10^4.1^, respectively. Then, the same method was used to prepare a flexible storage device on a polyethylene terephthalate (PET) substrate. The Al/Ti_3_C_2_T_*x*_‐OP MXene/PET device maintained good distinguishable multilevel storage characteristics even after 5000 bending cycles (Figure 9k,l) at a radius of curvature *ρ* of 2.1 cm. The findings of this study showed that MXene‐based composite materials are excellent candidates for flexible nonvolatile memory devices and are of great significance for the development and application of flexible electronic devices. The incorporation of MXene composites in RRAM proved that the surface functional groups and metal vacancies of Ti_3_C_2_ nanosheets are conducive to charge trapping, thereby trapping charge carriers and forming CFs when performing sweeping voltage, facilitating the HRS‐to‐LRS transition of RRAM. On the contrary, the LRS‐to‐HRS transition is due to the rupture of the CFs. Overall, MXenes have been designed for flexible and multilevel storage applications to overcome the shortcomings of traditional metal oxide RS layers. This has accelerated the research on the application of 2D materials in memory.

**Figure 10:**
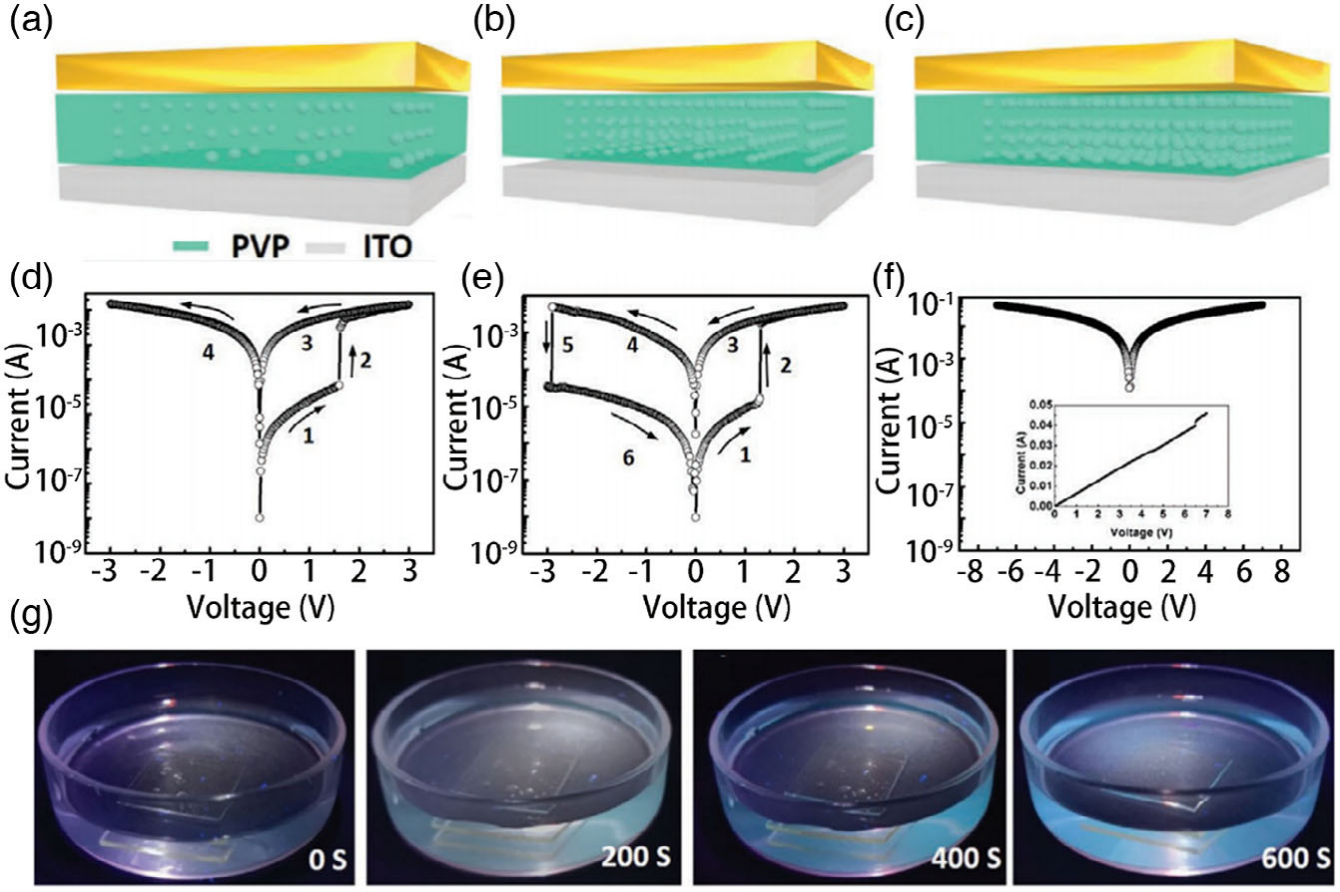
*I*–*V* characteristic curve of ITO/MQDs‐PVPy/Au device under different MQDs doping concentrations. When the doping concentration of MQDs in PVPy is a) 380 μg mL^−1^, b) 1.14 mg mL^−1^, and c) 3.42 mg mL^−1^, the corresponding *I*–*V* characteristic curve is (d)–(f). g) Schematic diagram of device degradation process. The optical image shows the degradation process from 0 to 600 s under ultraviolet light (365 nm). a–g) Reproduced with permission.^[21c]^ Copyright 2019, Wiley‐VCH.

Over the past few years, a series of novel quantum dots (QDs) such as graphene QDs and black phosphorous QDs derived from 2D materials have gained widespread attention. As a new type of 2D material, MXene quantum dots (MQDs) disperse well in water‐soluble polymers for charge trapping, thereby improving the reliability of data storage. In 2019, Mao et al. reported for the first time, an ITO/MQDs‐PVPy/Au storage device based on a MQDs/PVPy composite film as the RS layer.^[21c]^ In this study, the doping content of MQDs in PVPy was adjusted to precisely tune the nonvolatile storage behavior of the resulting device. Moreover, the effect of the MQD concentration on the electrical behavior of the resulting device was investigated. When the doping concentration of MQDs was 380 μg mL^−1^ and a positive voltage was applied to the device, the conductance state of the device changed because of the filling of the charge trapping center provided by the MQDs. However, when the bias voltage was annulled or when the reverse bias was applied, the trapped electrons were not released because of the built‐in electric field and the conductance state did not change. Therefore, the device exhibited the WORM behavior (**Figure** **10**a,d). When the MQD doping concentration was 1.14 mg mL^−1^ and the sweeping voltage was increased, the quantitative competition between the injected carriers and free carriers generated by the thermal excitation was the key reason for the device current transformation. When a negative electric field was applied, the contact between the MQDs and the electrode interface neutralized the charge carriers, and the device returned to the HRS, showing flash memory characteristics (Figure 10b,e). With a further increase in the MQD doping concentration to 3.42 mg mL^−1^, the device exhibited a conduction behavior similar to that exhibited by metals (Figure 10c,f). In addition, this study showed that memory devices based on MQDs‐PVPy materials exhibit remarkable physical transient characteristics. This study provided a new perspective for the development of green electronic devices based on MXene materials. As shown in Figure 10g, when the storage device based on MQDs‐PVPy was immersed in deionized water at room temperature, the active film disappeared completely after 600 s. In summary, compared with MXene nanosheets, MQDs are a safe and reliable RS material for emerging nonvolatile data storage due to their strong quantum confinement, edge effect, and hydrophilicity.

### 4.3 Artificial Neurons and Artificial Synapses

**Figure 11:**
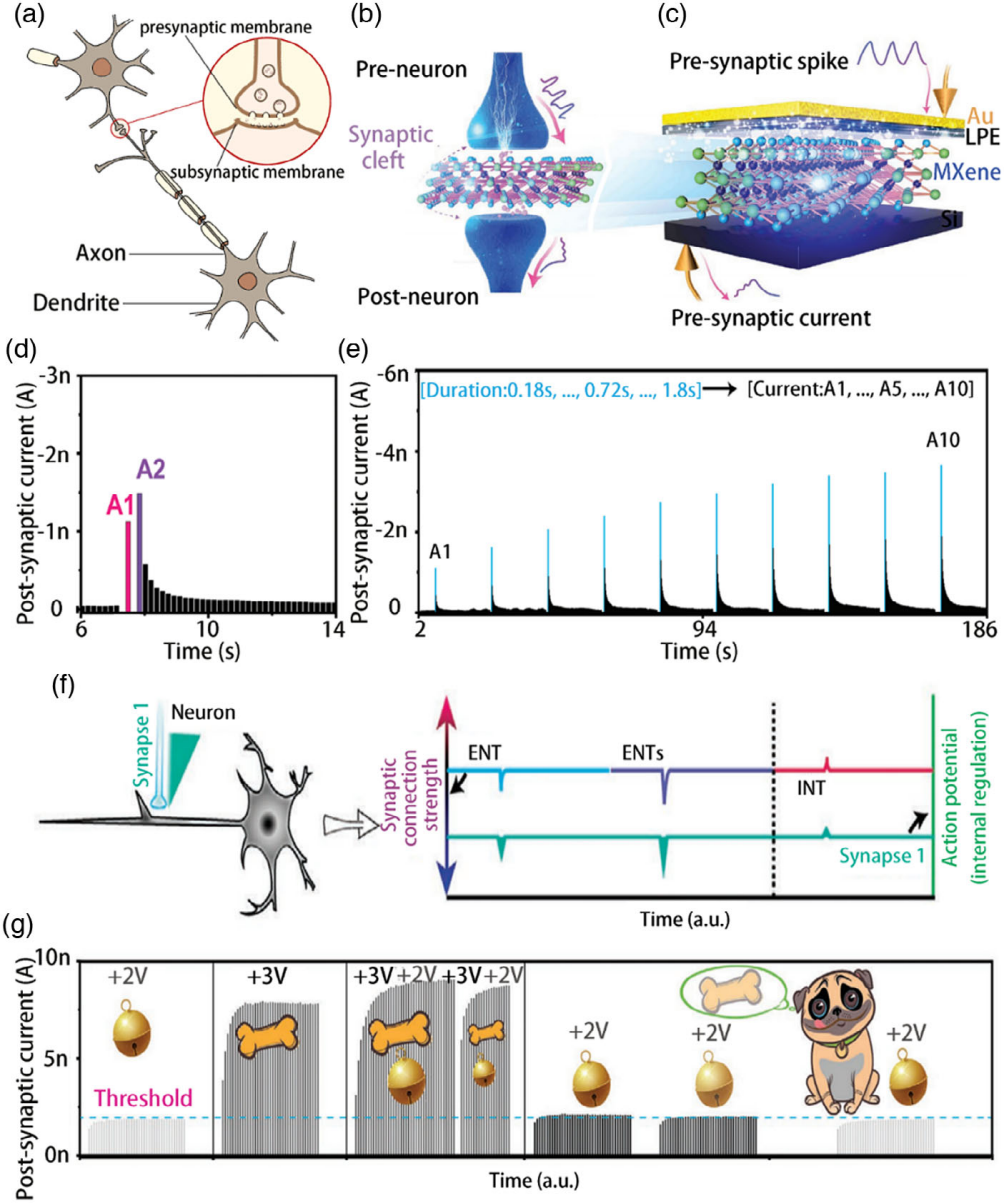
a) Schematic diagram of neurons and synapses. b) Schematic illustration of biological synapse and c) Ti_3_C_2_‐MXene artificial synapses. d) EPSC peak triggered by two successive negative spikes. e) spike‐duration‐dependent plasticity (SDDP) is clearly strengthened when the pulse duration is extended from 0.18 to 1.8 s. f) Bidirectional synaptic plasticity achieved by sequentially applying pulses with opposite polarity in MXene artificial neuron. g) Emulation of Pavlov's learning by dual presynaptic inputs. a–g) Reproduced with permission.^[^ ^57^ ^]^ Copyright 2021, Wiley‐VCH.

As mentioned earlier, by solving the problem of separation of storage and computing, the Von Neumann bottleneck can be overcome.^[^ ^54^ ^]^ However, the human brain can well establish the integration of storage and computing.^[^ ^55^ ^]^ This is because the human brain has a huge nervous system that can process and store complex information.^[^ ^56^ ^]^ Neurons are the basic structural and functional units of the nervous system and perform the function of connecting and integrating input information and transmitting output information. Synapses are the regions where neurons are functionally connected, and they also form a key part of information transmission (**Figure** **11**a).^[^ ^57^ ^]^ The first artificial simulation of neurons can be traced back to the 1950s, when researchers used vacuum tubes to simulate a network of 40 neurons and tried to build an artificial neural machine called spatial–numerical association of response codes (SNARCs). Since then, the simulation of biological neurons and synapses has been extensively studied.^[^ ^58^ ^]^ However, CMOS integrated circuits used for simulation usually have a sundry circuit architecture, which is unlikely to be the ultimate solution for future artificial intelligence applications in terms of power consumption and size. Meanwhile, due to their conduction mechanism, which is similar to neural dynamics, and structure, which is similar to that of synapses, memristors are the most reported nonlinear storage devices used to simulate artificial synapses and artificial neurons.^[^ ^59^ ^]^

A synapse is composed of a presynaptic membrane, a postsynaptic membrane, and the synaptic gap (the narrow gap between the two membranes). It can realize the transmission, processing, and integration of a large amount of parallel information by adjusting the synaptic weight while providing complex space and energy trade‐offs.^[^ ^60^ ^]^ Inspired by biological synapses, several artificial synapses have been developed. Nevertheless, there are two major challenges in the development of artificial synapses. First, to avoid energy dissipation or the refreshing of the memory state, the nonvolatile characteristics of memory are indispensable. Second, the functional materials used in artificial synaptic devices should exhibit the same response to external electrical stimuli, so that a relationship between the input stimuli and synaptic responses can be established. Based on this, Wei et al. reported a synaptic device coupled with a Ti_3_C_2_ film and a solid lithium–polymer–electrolyte (LPE) layer. The device exhibited paired‐pulse facilitation (PPF), spike‐rate‐dependent plasticity, and functions such as dendritic integration and memory enhancement.^[^ ^61^ ^]^ As shown in Figure 11b,c, the Au electrode and LPE layer together acted as the presynaptic membrane. The diffusion and transport of Li^+^ in the solid electrolyte were similar to the release and transmission of neurotransmitters, and the Ti_3_C_2_ membrane and Si substrate together played the role of the postsynaptic film. The biological synaptic plasticity is an important parameter affecting the ability of synapses to achieve memory and learning. It is a measure of the synapse strength (corresponding to conductance of the device) as a function of the activity of neurons (electrical signals). Depending on the length of pulse duration, synaptic plasticity can be divided into short‐term plasticity and long‐term plasticity (STP and LTP). In the Ti_3_C_2_ artificial synapses, when a pair of adjacent spikes was applied, the postsynaptic current increased, and the depolarization‐induced excitatory postsynaptic current increased sharply and then decreased in a short time, which is STP (Figure 11d). PPF is a polymerized form of STP. That is, in an artificial synapse, the application of one pulse will cause some ions to remain in the synapse, and then the second pulse will enhance the synaptic response. Similar to the behavior of biological synapses, artificial synapses can realize tunable synaptic weights by adjusting the pulse duration, number, amplitude, and frequency. Figure 11e shows the spike‐duration‐dependent plasticity of the Ti_3_C_2_ artificial synapses. When the spike amplitude was fixed, the increase in the pulse time increased the number of ions that escaped from the surface of the MXene, resulting in a significant increase in the synaptic strength. By the same principle, when the number of spikes increased from 1 to 10, the synaptic weight of the Ti_3_C_2_ synapses increased sharply, exhibiting spike‐number‐dependent plasticity. However, a continuous increase in the number of pulses (≈50) caused the synapse weight to reach saturation and completed the STP‐to‐LTP conversion process. This is because as the number of spikes increased, a significant number of Li^+^ ions migrated to the Ti_3_C_2_‐MXene/LPE surface, and some ions even escaped from the internal channel of Ti_3_C_2_. When the pulses were removed, the time for Li^+^ ions to return to their original position became longer, which means that the synaptic weight of Ti_3_C_2_ could be precisely adjusted by controlling the interval and sequence between the voltage pulses.

Information exchange by receiving, integrating, transmitting, and outputting information is the basic function of neurons. Wei et al. simulated the spatiotemporal relationship between the neuron action potential inputs by constructing two axon ends and a single dendritic end. The variation of the time interval (Δ*t*) of the neurotransmitter released by the two axon ends affected the response of the dendritic ends. This observation is of great significance for the complete simulation of biological synapses and the parallel transmission of information. Sensory neurons can release different types of neurotransmitters (excitatory and inhibitory neurotransmitters) when perceiving the external environment to regulate emotions. As shown in Figure 11f, by applying pulses of opposite polarity or different amplitudes, the MXene artificial neurons achieved bidirectional synaptic plasticity, indicating that the MXene artificial neurons exhibited a self‐regulating function. In addition, the authors simulated Pavlov learning by carrying out food (+3 V) and ringtone (+2 V) stimulation on the device. Relying only on the stimulus of the bells could make the puppy produce saliva, which can be attributed to the electrochemical doping of ions under pulse action (Figure 11g).

**Figure 12:**
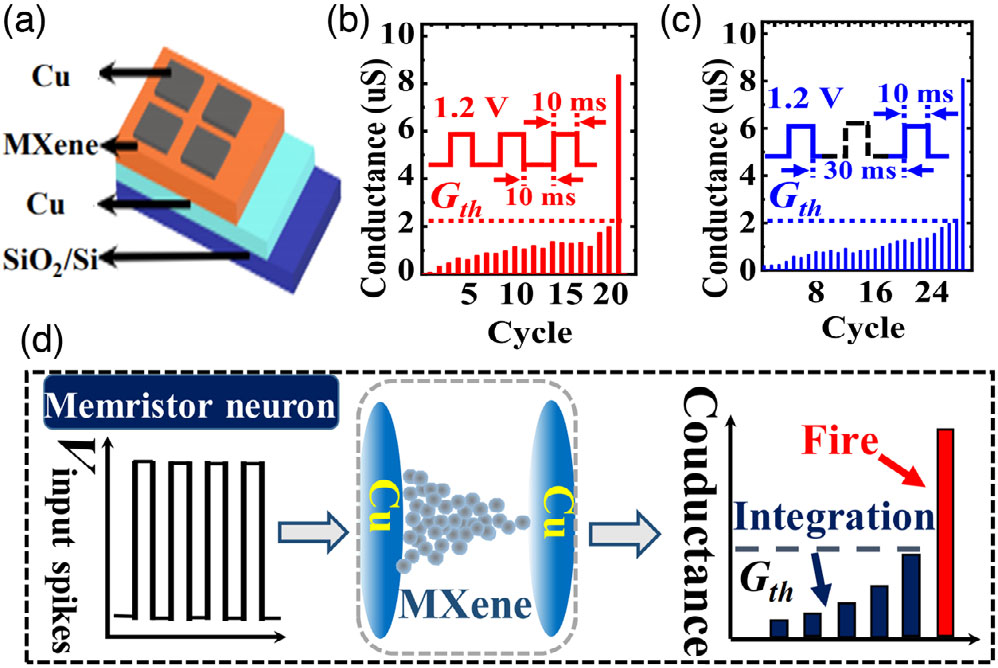
a) Schematic illustration of the structure of Cu/MXene/Cu memristor. b) A succession of 1.2 V square wave with a pulse width of 10 ms and a pulse interval of 10 ms was applied to the device. c) The pulse train with a pulse width of 10 ms and a pulse interval of 30 ms are applied to memristors to fire the artificial neuron. d) The conduction mechanism of Cu/MXene/Cu memristors and the simulation of leaky integrate‐and‐fire dynamics under a succession of pulses. a–d) Reproduced with permission.^[^ ^62^ ^]^ Copyright 2019, IEEE.

In addition to the basic characteristics of synapses and neurons, the neural processing system contains several essential contents, such as leaky integration, automatic threshold‐driven fire, and self‐recovery. In 2019, Chen et al. simulated various neural properties using RRAM with the Cu/MXene/Cu structure (**Figure** **12**a).^[^ ^62^ ^]^ In this report, the simulation of neurons did not require auxiliary circuits, and all the simulations were completed by a single MXene RS layer‐based RRAM. Figure 12d shows the simulation of biological integration‐and‐fire. The movement of Cu^+^ in MXene can be regarded as transmitter transmission in the ion channel of the neuron under the action of an external electric field. In the simulation of the “leaky” integration function, continuous pulses with an amplitude of 1.2 V and a pulse width and pulse interval of 10 ms were applied to the memristor. The results showed that the conductance of the device first increased gradually and then increased sharply at the 20th pulse (*G*_th_ ≈ 2 μs) (Figure 12b). The authors then applied a pulse sequence with an amplitude of 1.2 V, a pulse width of 10 ms, and pulse interval of 30 ms to the memristor. In contrast with previously reported results, the conductance was emitted at the 28th pulse in this study, showing a significant leakage effect (Figure 12c). It is worth mentioning that the power efficiency of the device was expected to be 8 μW, which is a great performance for a single device that simulates neuron activity. This work will provide useful guidance for the development of high‐density neuromorphic devices for single‐device simulation of neurons.

### 4.4 MXene‐based Logic Circuit

**Figure 13:**
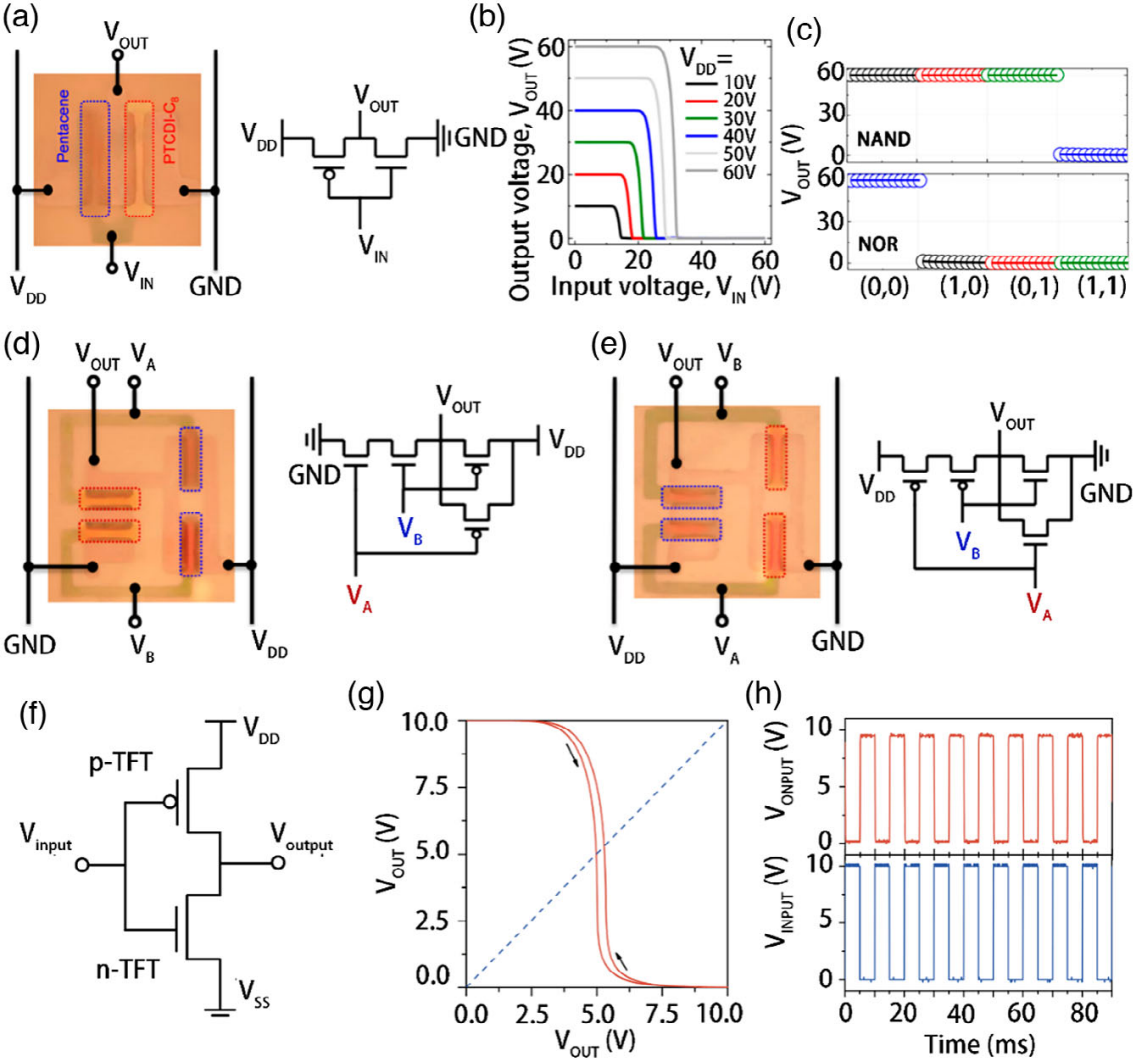
Optical top‐view image of fabricated a) NOT, d) NAND, and e) NOR gates and corresponding circuit diagrams. b) Voltage transfer characteristics of c) NOT gate. The output signals of the NAND and NOR gates. f) Schematic circuit diagram of the CMOS inverter with all‐MXene contacts. g) The VTC of this CMOS inverter. h) Dynamic response of the CMOS inverter to a 100 Hz square waveform is used as input. a–e) Reproduced with permission.^[^ ^46^ ^]^ Copyright 2019, American Chemical Society. f–g) Reproduced with permission.^[^ ^39^ ^]^ Copyright 2018, Wiley‐VCH.

Component efficiency has been rapidly enhanced and realized with the growth of the 2D material transistor manufacturing technology. The manufacture of simple 2D logic circuits has been reported. In integrated logic circuits, ultrathin 2D materials have great potential to expand the Moore's Law, but FETs are the main components incorporated in logic circuits. Ideally, 2D transistors should exhibit a high on/off current ratio to achieve low dynamic power consumption. However, the development of integrated logic circuits is still limited because of serious challenges such as voltage loss and high power consumption. It is important to explore new doping technologies to achieve the precise spatial regulation of doping levels for the production of logic circuits. For multifunctional electronic devices, the latest breakthroughs in the deliberate charge doping of 2D materials at the nanoscale have provided several opportunities. According to the Schottky–Mott rule, the carrier injection barrier can be determined from the difference between the WF of the electrode and the electron affinity of the semiconductor (or vacuum ionization energy). Thus, the wide WF tunability of MXenes can be attributed to the creation of Ohmic contacts, providing a wider range that can increase the efficiency of carrier injection. Using MXene electrodes in p‐type and n‐type FETs, as shown in **Figure** **13**a,d,e, Cho et al. manufactured complementary logic circuits on flexible substrates, including NOT, NAND, and NOR gates.^[^ ^46^ ^]^

The corresponding circuit diagram of the complementary NOT gate is shown in Figure 13a. The p‐type and n‐type FETs were linked in sequence, and the input and output terminals were shared. The voltage transfer features of the NOT gate and the corresponding signal gain (*V*_OUT_/*V*_IN_) are shown in Figure 13b. When the input voltage was 0 V, the voltage (*V*_DD_) applied to the drain electrode was the calculated output voltage. By assembling two p‐type and two n‐type OFETs with MXene electrodes, more complex logic circuits, such as NAND and NOR gates were fabricated. The top‐view optical images of the NAND and NOR gates and the corresponding circuit diagrams are shown in Figure 13d,e, respectively. Four possible logical combinations (0,0), (1,0), (0,1), and (1,1) were used to obtain the output signals for the NAND and NOR gates, and the voltage was +60 V, as shown in Figure 13c. In the case of the NAND gate, the three logic combinations (0, 0), (1, 0), and (0, 1) realized the logic state “1.” Only the (1, 1) logic combination reached the logical state “0.” In the case of the NOR gate, the logic state “1” was realized only by the logic combination (0, 0), whereas the other three logic combinations corresponded to the logic state “0.” The balanced performance of the n‐type and p‐type TFTs showed that it is possible to generate a high‐performance CMOS inverter with MXene contacts.^[^ ^39^ ^]^ Figure 13f shows the circuit diagram of a CMOS inverter with complete MXene contacts. Two transistors of thin films share a similar electrode gate (input terminal). The source of the p‐type TFT was used as the *V*_DD_ (power supply voltage) terminal. The n‐type TFT source acted as the terminal of the *V*_SS_ and was grounded. By linking the drains of the two TFTs, the output terminal was constructed. The p‐channel turned “on” and the n‐channel turned “off” when a small *V*_input_ was applied. This indicates that the *V*_output_ was equivalent to the *V*_DD_ (high state). With an increase in *V*_input_, the p‐channel turned “off” gradually, whereas the n‐channel turned “on,” resulting in a dramatic decrease in *V*_output_ (transition state). Finally, when *V*_input_ was sufficiently high, the n‐channel turned “on” and *V*_output_ became equal to *V*_SS_ (low state). The voltage transfer curve (VTC) of the semiconductor inverter of the complementary metal oxide is shown in Figure 13g. A dynamic stable operation under a 100 Hz square wave input was demonstrated by the CMOS inverter (Figure 13h). These findings showed that MXene (Ti_3_C_2_) films can be used as contacts for different electronic applications.

## 5 Conclusion

It is well‐known that 2D transition metal carbides, carbonitrides, and nitrides exhibit attractive electrical, optical, electrochemical, and mechanical properties and are used in various applications. A number of transition metals and surface terminations provide infinite possibilities for modifying the composition and performance of MXenes. This Review discusses the properties and recent advances in the electronic storage applications of 2D transition metal carbides and nitrides, as well as the fascinating tunable properties of MXenes, which can be modified by controlling their structure, transition metal elements, surface functional groups, and etching chemistry. These factors are related to each other and affect the properties of MXenes directly. The MXene family finds applications not only in the electrochemical field, but also in MXetronics. The key approaches for synthesizing MXenes are the top‐down and bottom‐up synthesis techniques. After the synthesis of MXenes, it is necessary to use the peeling method to obtain single‐layer MXenes. The electrical properties of MXenes and their possible use in storage applications are then discussed. For example, the energy bandgap of single‐layer MXenes can be strain‐modulated, and hence exhibits unique characteristics. Moreover, MXenes exhibit an adjustable WF. The second part of this Review discusses the advancements in the application of MXenes in memory devices. MXenes show huge potential for application in transistor electrodes and channels because of their excellent energy bandgap and fairly good carrier mobility. The progress in artificial synapses and logic circuits using MXenes has also been discussed, and the structure, memory characteristics, and working mechanism of MXene‐based RRAM have been elaborated. Whether used as a resistive layer or an electrode, MXenes have made great breakthroughs in terms of high switching speed and good durability.

As promising candidates for nonvolatile memory devices, MXene‐based composite materials are of great significance for the production and commercialization of flexible electronic devices. MXene‐type synapse‐driven devices can be classified as synaptic devices of two‐terminal memristors and three‐terminal FET‐type devices, which can realistically simulate the plasticity‐dependent spike timing and learning/forgetting mechanism of the biological nervous system. The elastic and plastic roles of synapses can be attributed to the information memory and learning/forgetting capacity of the biological nervous system. Research on memory‐based synaptic devices therefore focuses primarily on realizing the processing of parallel information. Future research on these individual materials and their heterogeneous structures will bring about substantial scientific discoveries and technical developments. We hope this Review will serve as a guide to understand the electronic properties of MXenes and will help bridge the gap between theoretical predictions and experimental observations. We want to emphasize that the knowledge on MXenes should be combined with the experience gained from the basic electronic applications of graphene, TMDs, and other 2D materials to realize the full potential of MXenes for electronic applications. It is also time to increase our understanding of the features of MXenes to realize efficient storage applications of this 2D material family.

## Conflict of Interest

The authors declare no conflict of interest.
